# The physiology of MASLD: molecular pathways between liver and adipose tissues

**DOI:** 10.1042/CS20257571

**Published:** 2025-09-22

**Authors:** Wang-Hsin Lee, Zachary A. Kipp, Evelyn A. Bates, Sally N. Pauss, Genesee J. Martinez, Terry D. Hinds

**Affiliations:** 1 Drug & Disease Discovery D3 Research Center, Department of Pharmacology and Nutritional Sciences, University of Kentucky College of Medicine, Lexington, KY, U.S.A; 2 Barnstable Brown Diabetes Center, University of Kentucky College of Medicine, Lexington, KY, U.S.A; 3 Markey Cancer Center, University of Kentucky, Lexington, KY, U.S.A

**Keywords:** bile acids, bilirubin, cytokines, fibrosis, obesity, oxidative stress

## Abstract

The global prevalence of obesity has exerted a profound influence on human health. It has contributed to numerous obesity-related comorbidities, including metabolic dysfunction-associated steatotic liver disease (MASLD) and insulin-resistant diabetes. MASLD is diagnosed when there is substantial fat accumulation concomitant with five additional diagnostic criteria. If untreated, MASLD may progress to liver fibrosis and cirrhosis, conditions that can be life-threatening in the final stages. Nonetheless, the development and progression of MASLD are complex, and its underlying mechanisms remain incompletely elucidated. Typically, during fasting, adipose tissue releases fatty acids, which the liver subsequently uptakes for gluconeogenesis. However, this process, along with many others, is impaired in the liver and adipose tissue of individuals with MASLD. This review provides comprehensive details on the mechanisms underlying adiposity and insulin resistance associated with MASLD. We discuss the canonical pathways that promote lipogenesis and insulin sensitivity in the liver and adipose tissues, including bile acids, bilirubin, fatty acids, inflammation, *de novo* lipogenesis, oxidative stress, peroxisome proliferator-activated receptors (PPARs), fibroblast growth factor 21 (FGF21), glucagon-like peptide 1 (GLP1), and metabolism of fructose. The scope of the review is expanded to encompass biological responses to fasting and feeding, as well as their effects on fat accumulation and insulin sensitivity in these tissues. Additionally, the review elaborates on critical molecular mechanisms regulating MASLD progression, including hepatic insulin clearance, insulin degradation, bilirubin metabolism, nerve innervation, and the roles of cytokines and adipokines. Overall, this review examines the mechanisms driving MASLD and explores potential novel therapeutic strategies for its management.

## Introduction

When fat accumulates in the liver, often as a consequence of diets high in calories and an imbalance between energy intake and expenditure, it serves as a catalyst for insulin resistance [1]. Individuals with elevated body mass indices (BMIs) exceeding 30 are predisposed to develop metabolic dysfunction-associated steatotic liver disease (MASLD), which is estimated to affect nearly one-third of adults globally [2]. The worldwide prevalence of MASLD in 2021 was 1.27 billion individuals [3]. MASLD grading is characterized by the extent of hepatic fat accumulation, as outlined in Table 1 [4], determined through imaging or biopsy. The diagnosis of MASLD is established when there is significant fat accumulation along with at least one of the five criteria detailed in Table 2 [5]. Most therapeutic interventions for MASLD aim to reverse the insulin resistance associated with the condition, which is discussed extensively throughout. If left untreated, MASLD has the potential to progress to severe complications such as hepatic fibrosis (referred to as metabolic dysfunction-associated steatohepatitis, MASH), cirrhosis, and hepatocellular carcinoma [1]. Resmetirom, a thyroid receptor-β agonist, is the only medication FDA-approved for the treatment of MASH with liver fibrosis (approved for stages F2/F3) [6]. More investigations are needed to improve the scientific understanding of these processes and how to reverse them.

**Table 1:** MASLD Grade of Liver Disease and Fat Percentage

Grade	Percent fat	Liver condition
**0**	0–3%	Normal
**0**	<5%	Simple steatosis
**1**	5–33%	Mild
**2**	34–66%	Moderate
**3**	>66%	Severe

MASLD, metabolic dysfunction-associated steatotic liver disease.

**Table 2:** Criteria for MASLD Diagnosis

Five additional criteria for diagnosis of MASLD		
	Diagnostic test	Range of characteristics
**1**	Body mass index (BMI)	≥25 kg/m^2^ ( ≥ 23 kg/m^2^ in Asians) or waist circumference >94 cm in men, >80 cm in women or ethnicity-adjusted
**2**	Fasting serum glucose	≥100 mg/dl Or HbA1c ≥5.7%
**3**	Blood pressure	≥130/85 mmHg
**4**	Plasma triglycerides	≥150 mg/dl (≥1.70 mmol/l)
**5**	Plasma HDL cholesterol	<40 mg/dl (< 1.0 mmol/l) for men and <50 mg/dl for women

HDL, high-density lipoprotein. MASLD, metabolic dysfunction-associated steatotic liver disease.

MASLD and insulin resistance have emerged as significant concerns affecting human health globally. The prevalence of insulin resistance varies internationally, with rates ranging from 15% to 47% [7–9]. In the United States, approximately 20% of adults are affected by insulin resistance [9], with certain states, such as Texas [10], reporting rates as high as 39.1% [8]. Data from the American Diabetes Association indicate that approximately 70% of individuals with insulin resistance eventually develop type 2 diabetes mellitus (T2DM) [11]. Patients with T2DM are often at risk of developing additional complications, including cardiovascular diseases and severe conditions such as stroke [12,13]. Unfortunately, the incidence of insulin resistance and T2DM continues to increase, with projections estimating a doubling of cases by 2050 [14].

Although the liver plays an indispensable role in regulating overall insulin sensitivity, insulin resistance does not originate from a single pathway or a singular organ. Instead, it represents a systemic disruption of metabolism involving multiple organs, tissues, and intertissue interactions. The primary insulin-sensitive organs responsible for glucose uptake are the muscles, adipose tissue, and liver. During both fasting and feeding states, the liver and adipose tissue engage in reciprocal cross-talk through lipoproteins originating from the liver that are subsequently taken up by adipose tissue, and vice versa. Notably, adipose tissue releases free fatty acids that are subsequently taken up by the liver. Concepts such as adipose tissue expandability and the multihit liver hypothesis address the interrelationships between the liver and adipose tissue in the context of fat accumulation and insulin resistance [15,16].

While these signaling mechanisms contribute to the development of MASLD, other essential factors, although not discussed further, include the gut–liver axis, microbiome, and epigenetics, all of which have shown potential involvement in the progression of hepatic fat accumulation.

Herein, we focus the discussion on the physiological signaling processes between the liver and adipose tissues that govern insulin sensitivity and metabolic conditions of MASLD. We discuss the mechanisms of key regulators in the development of MASLD, including adipose-derived hormones and cytokines, carcinoembryonic antigen cell adhesion molecule 1 (CEACAM1), peroxisome proliferator-activated receptor alpha (PPARα), PPARγ, farnesoid X receptor (FXR), as well as other factors such as neural elements that modulate the adipose–liver axis. A comprehensive understanding of the interrelationships between the liver and adipose tissues may reveal the underlying molecular mechanisms that lead to insulin resistance, which is essential for the development of more effective therapeutic strategies against MASLD.

## Part 1: Metabolic signaling in the liver

### Lipoproteins and liver distribution to peripheral tissues

**Figure 1:**
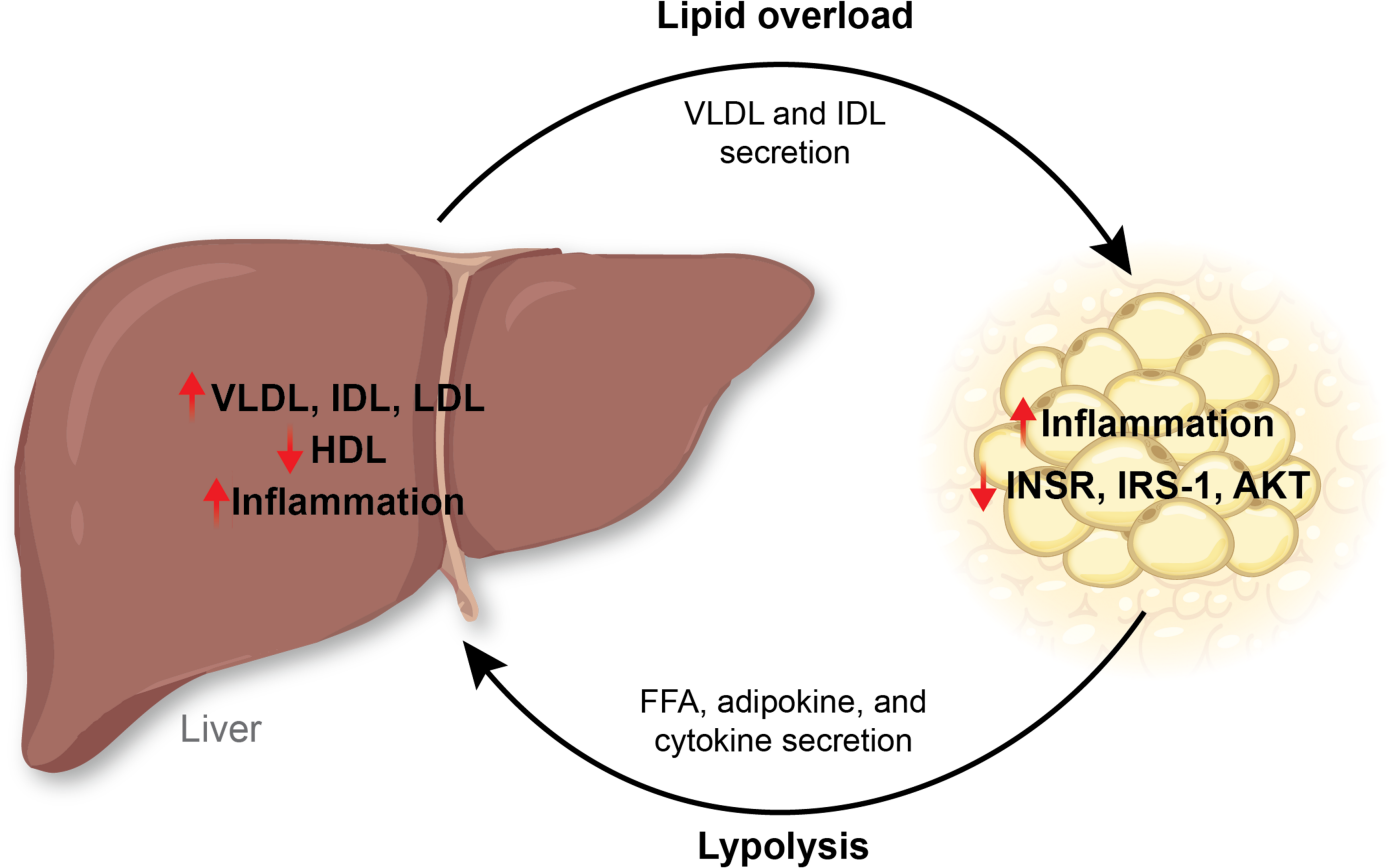
In an insulin-resistant state, homeostatic signaling mechanisms are disrupted between the adipose and the liver. The liver can secrete VLDL and IDL to transport lipids to adipose tissues. However, an overload of lipids in adipose tissues can result in adipose tissue inflammation and decrease the insulin receptor, IRS-1, and AKT activity. On the other hand, adipose tissues can release their lipid storage as free fatty acids when lipolysis activity increases. Adipose tissues also release adipokines and cytokines into the circulation, activating a pro-inflammatory signal in the liver. IDL, intermediate-density lipoprotein; IRS-1, insulin receptor substrate 1; VLDL, very low-density lipoprotein.

Lipoproteins such as very low-density lipoprotein (VLDL) carry triglycerides and cholesterol from the liver to the adipose (Figure 1). In an insulin-resistant state, there is a decrease in high-density lipoprotein (HDL) and an increase in VLDL [17,18]. Increases in VLDL in the liver in an insulin-resistant state are due to less efficient lipolysis of VLDL and increased VLDL secretion [19–21]. Donnelly et al. showed that patients with obesity and MASLD who also had fasting hyperinsulinemia and hypertriglyceridemia had 59.0% ± 9.9% of triacylglycerides that were derived from non-esterified fatty acids; 26.1% ± 6.7%, from *de novo* lipogenesis (DNL); and 14.9% ± 7.0%, from diet, and this was similar for VLDL [22]. Individuals with insulin resistance showed increased VLDL size and concentration, decreased LDL and small LDL particle concentrations, and decreased HDL size and concentration as determined by nuclear magnetic resonance (NMR) [23]. These data indicate that insulin resistance affects VLDL, HDL, and LDL size, as well as subparticle concentrations. Similarly, patients with MASLD had higher LDL concentrations and decreased LDL size [24]. Therefore, the lipoprotein landscape is altered in a hepatic insulin-resistant state, resulting in more pathological VLDL particles.

Other lipoproteins commonly dysregulated in insulin resistance are intermediate-density lipoproteins (IDL). Individuals with obesity have an accumulation of IDL in the adipose and liver, contributing to insulin signaling disruption [25]. IDL is generated after the metabolism of VLDL by lipoprotein lipase (LPL), predominantly in adipose and muscle tissues [26–28]. Women with gestational diabetes had an increase in VLDL and IDL [29]. Overproduction of LDL, IDL, and VLDL is common in an insulin-resistant state, and it is suggested that the liver also secretes IDL particles [30,31]. In summary, hepatic lipid accumulation and MASLD development are influenced by multiple factors, including DNL activity, oxidative stress, hepatic insulin clearance, fructose consumption, the activity of PPARs, and lipoprotein profile.

### Cross-talk in fasting and feeding responses

Fasting and feeding affect the cross-talk between adipose and liver tissues in different ways. During fasting, the liver maintains blood glucose levels through the processes of gluconeogenesis and glycogenolysis [1]. The pancreatic α-cells detect the reduced blood glucose and release glucagon into the circulation. The liver receives glucagon stimulation from the portal vein blood flow and releases glucose stored as glycogen due to glucagon-induced glycogenolysis [1]. Another process that regulates blood glucose levels is gluconeogenesis, which utilizes non-hexose substrates, such as fatty acids or amino acids, to generate sugars [32,33]. Factors such as the glucocorticoid receptor (GR), PPARα, and forkhead box protein O1 (FOXO1) have well-studied roles in regulating genes that control hepatic gluconeogenesis, including glucose-6-phosphatase (*G6PC*) and phosphoenolpyruvate carboxykinase 2 (*PCK2*), which are essential for releasing glucose into the circulation [33]. These processes restore blood glucose as an energy source for brain cells, red blood cells, and other tissues. However, in hepatic insulin resistance, gluconeogenesis is increased likely because of increased GR inhibitory proteins that block the negative feedback loop of glucocorticoids [33], such as the non-glucocorticoid binding isoform [34], GRβ [35], and others that bind to the GR steroid receptor complex, such as FKBP51 [36]. A hepatic loss of FKBP52, a positive regulator of the hormone-binding isoform, GRα [37], caused mice to have worsened MASLD characteristics when fed a diet high in fat compared with the control mice [38]. Mice with a global FKBP51 knockout were resistant to diet-induced obesity, MASLD, and insulin resistance [39]. These support that MASLD is a glucocorticoid-resistant state, which causes an accelerated production of ceramides in the liver [40]. Hepatic glucocorticoid resistance has been demonstrated by a study by Marino et al*.,* who showed that hepatic overexpression of GRβ induces lipid accumulation, inflammation, and glucose intolerance, which occurred by its suppression of PPARα [41]. GRβ levels are increased by insulin [42,43], and when there are high circulating insulin levels, GRβ expression is significantly higher [41]. PPARα mediates hepatic fatty acid storage by regulating genes for fat utilization, which we discuss further below.

**Figure 2:**
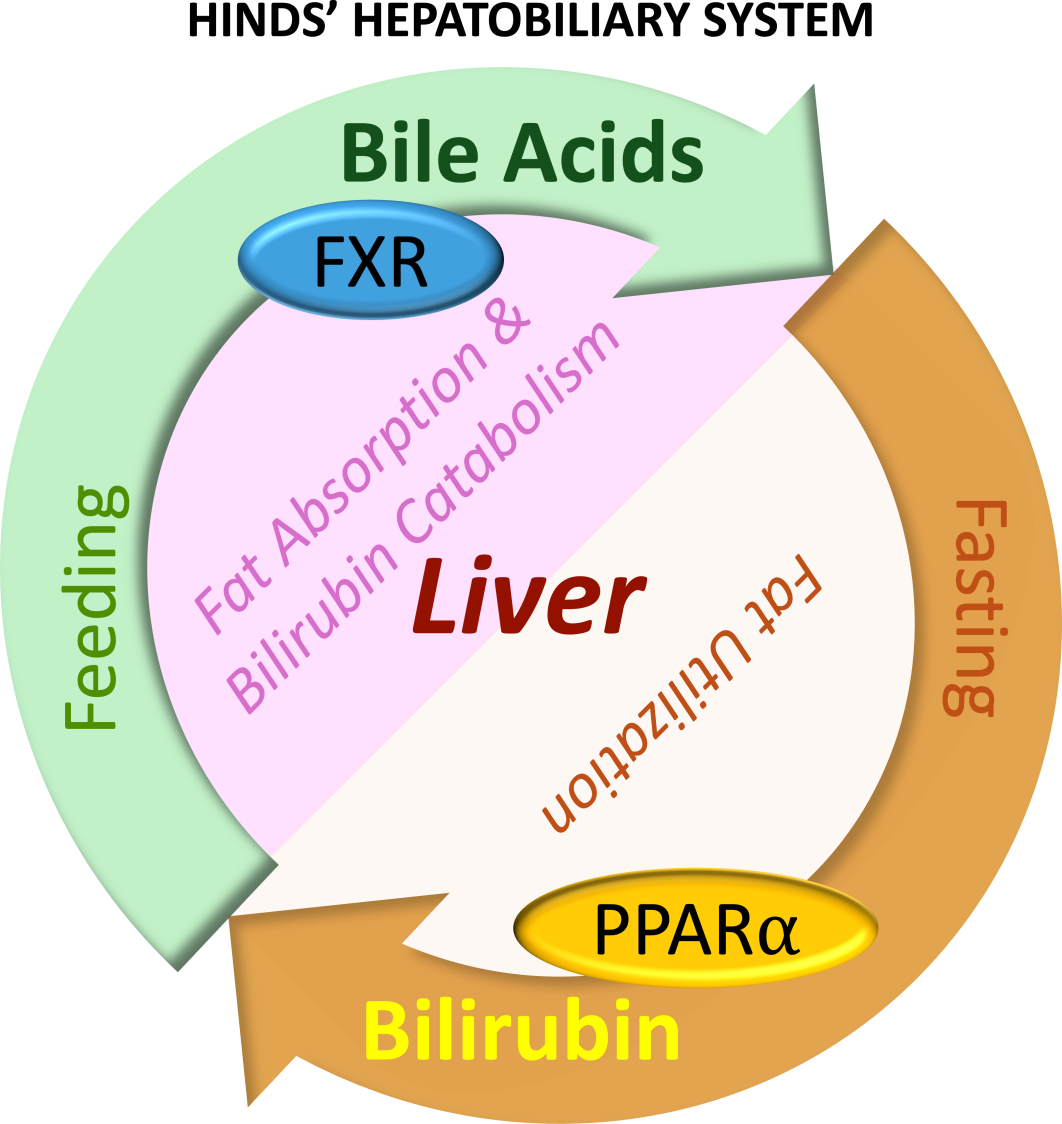
Biliary responses to fasting and feeding in the liver. Feeding increases hepatic bile acid production, activates hepatic FXR for fat absorption in the gut, and causes bilirubin catabolism. Fasting increases PPARα expression and bilirubin levels, which together stimulate fat utilization. FXR, farnesoid x receptor. PPARα, peroxisome proliferator-activated receptor alpha.

**Figure 3:**
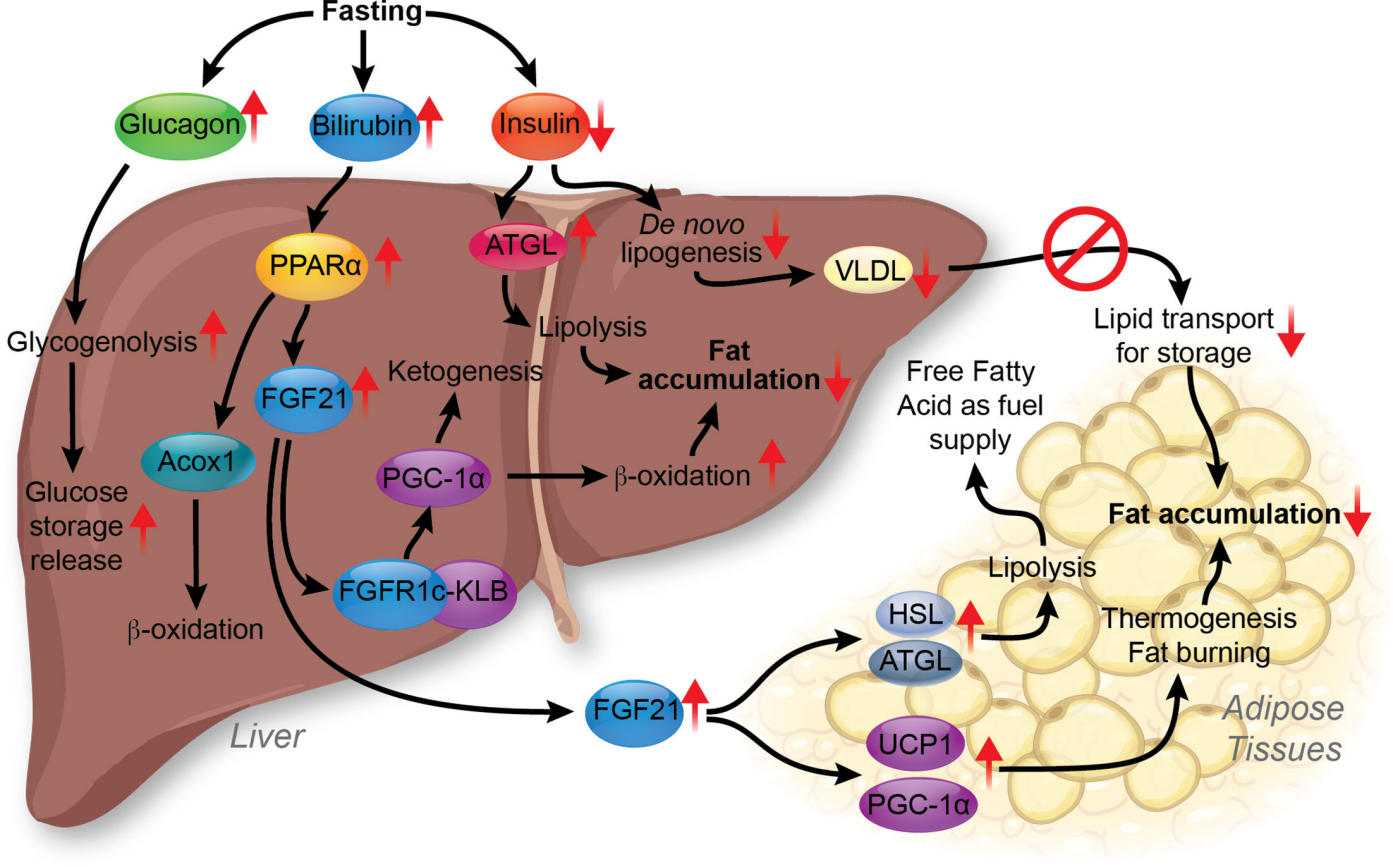
Molecular interactions between the liver and white adipose tissue in fasting. Fasting conditions lead to increased bilirubin, glucagon, and decreased insulin levels. Increased glucagon promotes glycogenolysis and glucose storage release. Higher bilirubin activates hepatic PPARα, which leads to FGF21 up-regulation. FGF21 can activate FGFR1c-KLB signaling in the liver, promoting β-oxidation and ketogenesis by increasing PGC-1α. Lowered insulin results in increased ATGL and lipolysis activity, which, combined with higher β-oxidation and ketogenesis, reduces hepatic fat accumulation. Lowered insulin inhibits liver *de novo* lipogenesis and VLDL production, suppressing lipid transport from the liver to adipose tissues. The FGF21 released from the liver can travel to adipose tissues through circulation, up-regulating HSL, ATGL, PGC-1α, and UCP1. The up-regulation of HSL and ATGL increases lipolysis in the adipose tissue, and FFA release is used to supply energy to other organs and tissues. The increase in PGC-1α and UCP1 promotes fat-burning and thermogenic effects, respectively. These effects lead to suppressed fat accumulation in the adipose tissues. FFA, free fatty acid; HSL, hormone-sensitive lipase; VLDL, very low-density lipoprotein.

The biliary system is essential in regulating lipid absorption and utilization during fasting and feeding (Figure 2). Bilirubin is elevated during fasting (and exercise [44–46]), and bile acids are involved in the absorption of fats during feeding. The latter is discussed further in the next section. Fasting increases PPARα expression and activity in the liver [47], which increases its hepatic target gene expression, fibroblast growth factor 21 (FGF21), and its release from the liver to the circulation [48]. PPARα is the liver’s most highly expressed PPAR isoform and drives fat-burning pathways to lower hepatic lipid content (Figure 3). Hepatic expression of PPARα is negatively correlated with the prevalence of liver steatosis and MASH [49]. The loss of PPARα in the liver caused hepatic lipid accumulation in chow-fed mice, which was worsened with high-fat feeding [50], and the hepatic loss of PPARα leads to cardiovascular disease [51]. PPARα is activated by endogenous ligands, including bilirubin and fatty acids, which directly bind to activate PPARα [52–54]. This binding recruits coactivator proteins to PPARα, leading to the transcription of its target genes [54]. In human hepatocytes, about 95% of the transcriptomic responses to bilirubin depend on the presence of PPARα [55]. Bilirubin is a strong and specific ligand for PPARα, which promotes fat-burning β-oxidation [53,54,56]. Increased bilirubin levels have also been demonstrated to reduce WAT size in diet-induced obese mice [54]. Scientific evidence suggests a negative correlation between serum bilirubin and MASLD risks [57,58], and that higher serum bilirubin levels reduce all-cause mortality and cardiovascular-associated mortality in MASLD [58,59].

Bilirubin functioning as a PPARα ligand may explain why mildly elevated plasma bilirubin is protective against MASLD and other metabolic disorders, including obesity and diabetes [58,60,61]; in animal models of MASLD, bilirubin nanoparticle treatment significantly reduced hepatic lipid accumulation through fatty acid β-oxidation activation via PPARα [56]. Bilirubin nanoparticles increased the hepatic expression of acyl-CoA oxidase 1 (ACOX1), leading to substantially higher β-hydroxybutyrate in the plasma, a ketone that indicates expedited fat burning [56]. Another hepatoprotective effect of bilirubin nanoparticles is to reduce the accumulation of ceramides in the liver [62]. Elevated hepatic ceramides are associated with steatosis, inflammation, and insulin resistance [40,63]. Bilirubin–PPARα axis increases hepatic expression and plasma levels of FGF21, as PPARα global knockout mice had no response to FGF21 induction with bilirubin, whereas the wildtype mice did show induction [53]. Bilirubin activation of PPARα increases carnitine palmitoyl-transferase 1A (CPT1A) and FGF21 expressions [64]. The increase in CPT1A and FGF21 promotes fatty acid oxidation, lipid metabolism, and insulin sensitivity [65,66].

FGF21 released from the liver triggers FGF receptor 1 c and 3 c (FGFR1c and FGFR3c) signaling in multiple organs and tissues, including the liver, brain, pancreas, and adipose [67]. In the liver, FGF21 binds to FGFR1c and forms a complex with β-Klotho (KLB), which increases proliferator-activated receptor gamma coactivator 1α (PGC-1α) expression [68,69]. This promotes hepatic β-oxidation and ketogenesis activities. Inagaki et al. demonstrated that FGF21 increases the expression of hormone-sensitive lipase (HSL) and adipose triglyceride lipase (ATGL) in adipose tissues and serum-free fatty acid (FFA) levels [70]. This indicates that FGF21 induces lipolysis in adipose tissue. The FGF21-mediated FFA release from adipose tissues provides fuel for hepatic β-oxidation and ketogenesis in fasting conditions [71]. FGF21 also stimulates the expression of uncoupling protein 1 (UCP1) and PGC-1α in adipose tissue, resulting in thermogenic and fat-burning effects [72]. The increased FGF21 helps the liver and adipose tissues utilize fat storage to fulfill the energy needs during fasting.

Elevated plasma FGF21 levels have been found in patients with MASLD and MASH and are considered a potential diagnostic marker of MASLD [67]. More studies are needed to understand the increased FGF21 levels observed, as it is regarded as a fat-burning hormone. From a clinical perspective, using FGF21 as a treatment agent has proven challenging due to its short half-life (0.5 to 2 hours) and rapid degradation [73]. To overcome this limitation, modified FGF21 analogs, such as efruxifermin and pegozafermin, have been developed to prolong their half-life and therapeutic effects [73]. Efruxifermin has demonstrated efficacy in reducing liver fat accumulation in clinical trials [73], while pegozafermin, a glyco-pegylated recombinant FGF21 analog, has also been shown to reduce liver fat content significantly [73]. Other FGF21 analogs, including BOS-580 and NNC0194-0499, are in the early stages of development, and more data are needed to understand their efficacy better [73].

In addition to PPARα and FGF21, insulin levels can affect lipid metabolism between the liver and adipose tissues [1]. Low insulin levels in the fasted state increase ATGL activity [74], stimulating lipolysis and triglyceride breakdown in adipose tissues. Without insulin stimulation, DNL decreases in hepatocytes [75]. This can lead to the formation of VLDL at a lower density and reduced lipid transport from hepatocytes to adipose tissues (Figure 3) [76]. In summary, fasting conditions stimulate FFA transport from white adipose tissue (WAT) to the liver to support the energy demand in higher β-oxidation and ketogenesis activities while suppressing lipid transport from the liver to the adipose tissue.

During feeding, blood glucose levels increase, which stimulates insulin secretion from pancreatic β-cells. Insulin increases glycogenesis and DNL in hepatocytes. Glycogenesis increases glucose storage as glycogen in the liver, whereas DNL increases fatty acid production in the liver. This leads to increased hepatic production of lipids and the release of VLDL into the circulation, by which the liver transports triglycerides and cholesterol to other tissues, such as adipose tissue. Insulin decreases VLDL production in the short term. However, prolonged exposure to high insulin stimulation can increase VLDL secretion [77,78].

#### Feeding response and bile acids

**Figure 4:**
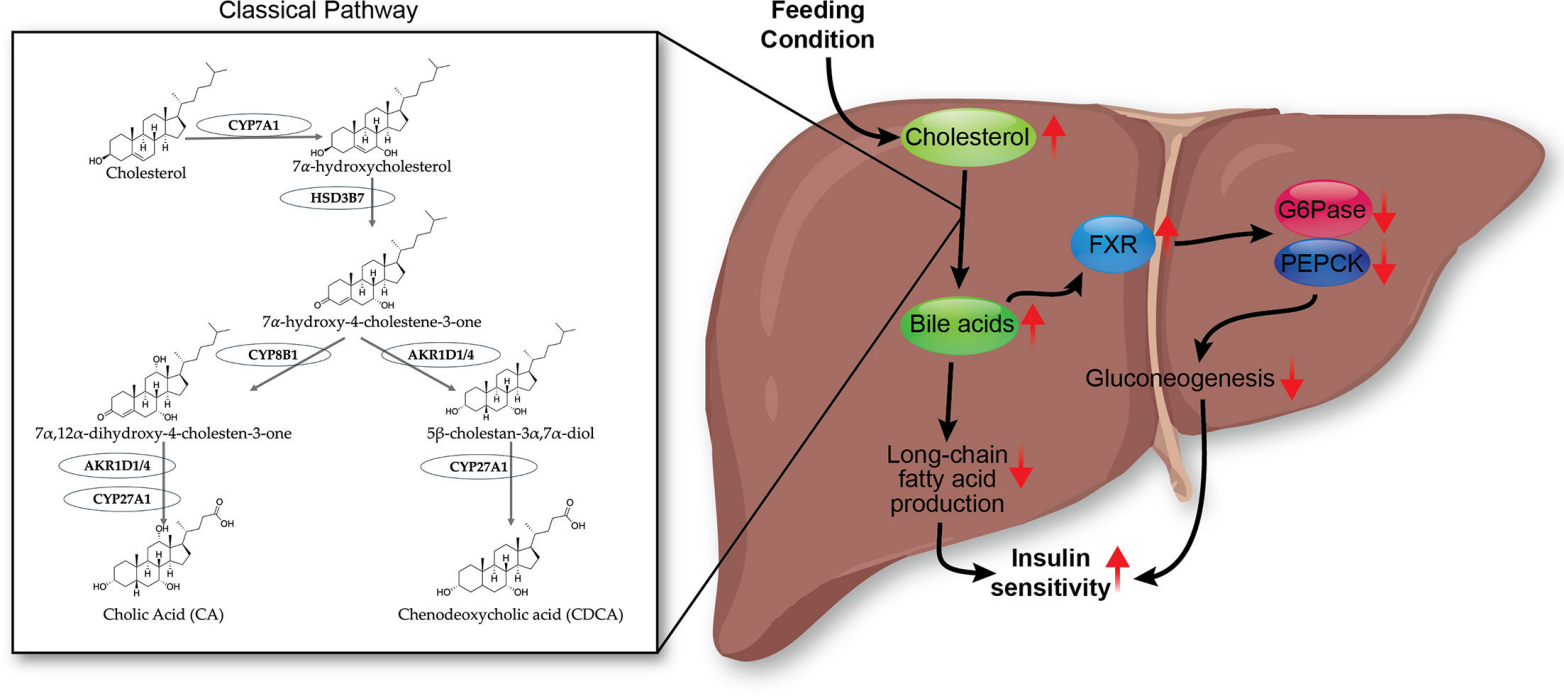
Bile acid-FXR pathway in the liver during feeding. Feeding or postprandial conditions increase hepatic cholesterol levels, stimulating bile acid production via the classical pathway through CYP7A1, HSD3B7, CYP8B1, AKR1D1/4, and CYP27A1. The increased bile acid pool activates hepatic FXR to suppress G6Pase, PEPCK, long-chain fatty acid production, and gluconeogenesis, which subsequently increases insulin sensitivity. FXR, farnesoid X receptor.

Bile acids are synthesized in the liver from cholesterol (Figure 4) [79]. Plasma bile acid concentrations increase postprandially [80], which then travel to the intestine via the biliary system and assist in the absorption of fats from the diet [81]. Bile acids, such as chenodeoxycholic acid (CDCA), deoxycholic acid (DCA), and cholic acid (CA), are endogenous ligands for the FXR nuclear receptor transcription factor [82–85], and their combined signaling regulates genes involved in lipid and glucose metabolism [86]. Activated FXR in hepatocytes increases small heterodimer partner (SHP) expression, reduces gluconeogenesis by inhibiting glucose-6-phosphatase (G6Pase) and phosphoenolpyruvate carboxykinase (PEPCK), and it also decreases *de novo* lipogenesis as discussed later [86,87]. Bile acid levels are closely associated with MASLD and insulin resistance through FXR signaling [88]. Bile acids, such as CDCA, DCA, and CA, induce FXR activation, which regulates fatty liver development and has been considered a potential treatment target for MASLD [89]. Suppressed FXR transcriptional signaling has been found in MASLD [90]. In addition, losing FXR has been reported to increase fat accumulation in the liver and circulating cholesterol and triglycerides [91,92]. Overall, the bile acid-induced FXR pathway decreases the production of fat and glucose in the liver and improves insulin sensitivity. However, it remains unclear whether bile acid-induced FXR signaling independently modulates MASLD phenotypes or as the disease progresses.

From a clinical perspective, FXR agonists, such as obeticholic acid (OCA), show potential as therapeutic agents for MASLD. A study has demonstrated that OCA treatment reduced fibrosis and steatosis in MASH patients [93]. However, the majority of FXR agonists activate FXR globally, which may lead to undesirable side effects such as pruritus [94]. Despite many being in phase II clinical trials, concerns remain regarding the FXR agonist’s adverse effects [94].

#### Fasting response and bilirubin

Bilirubin is a downstream metabolite from heme degradation that occurs when red blood cells lyse [61]. Bilirubin levels in the circulation are induced during fasting [95–98]. Bilirubin has long been considered a waste product cleared from the liver by the uridine diphosphate glucuronosyltransferase 1A1 (UGT1A1) enzyme [99] and a marker of liver damage; however, as described above, more evidence has demonstrated that bilirubin functions as a hormone by binding and activating PPARα [61,100]. The level of circulating bilirubin is regulated by hepatic UGT1A1 UDP-glucuronosyltransferase activity [99,101,102], which converts hydrophobic unconjugated bilirubin into a more hydrophilic conjugated form to promote its excretion through the biliary system [103]. Reduced UGT1A1 activity leads to unconjugated hyperbilirubinemia [89], which in the past has been viewed as pathological. However, patients with Gilbert’s syndrome, caused by a polymorphism in the *UGT1A1* gene promoter that results in its protein with reduced activity, have a 2–3 fold increase in plasma bilirubin levels and lower cardiovascular risks [104]. These indicate a strong connection between liver bilirubin metabolism and other tissues’ metabolic regulation [58]. A mildly reduced hepatic bilirubin excretion paired with a two- to three-fold increase in bilirubin levels inhibits fat accumulation in tissues like the liver and adipose.

When excreted into the gut, bilirubin is converted into urobilin through gut bacterial activities mediated by the bilirubin reductase (BilR) bacterial enzyme [58,105]. Urobilin can be absorbed via the hepatic portal vein to enter circulation [58]. Notably, urobilin has been reported to be increased in the plasma of people with obesity [106], and this probably elevates the risks of cardiovascular disease and insulin resistance. Urobilin levels have also been reported to be lower in humans and rodents that are lean compared with their obese counterparts [106]. Additionally, urobilin levels are positively correlated with higher HOMA-IR in both men and women [106]. These findings suggest that urobilin has an adverse effect on insulin sensitivity and adiposity [58]. However, the mechanism by which urobilin regulates fat accumulation and insulin resistance is still unknown. Urobilin has only been shown to bind to albumin [107], and its signaling mechanisms are currently unknown.

#### Fatty acids in the liver–adipose axis

Consumption of a diet high in fat exacerbates the development of MASLD [108,109]. Imbalances in saturated and unsaturated fatty acids, omega-6 and omega-3, can affect inflammation and lipid accumulation in the progression of MASLD and insulin resistance. Metabolized omega-6 produces pro-inflammatory compounds, and omega-3 compounds are anti-inflammatory [110]. Both omega-6 and omega-3 are metabolized by cyclooxygenase (COX) to produce prostaglandins (PG). Omega-6 fatty acids are converted into the PGE2, while omega-3 fatty acids become the anti-inflammatory PGE3. PGE2 is most often associated with proinflammatory signaling actions; however, studies have also indicated that it may have anti-inflammatory roles [111]. Bagga et al. demonstrated that PGE2 induces higher COX-2 gene expression in NIH 3T3 cells and interleukin-6 (IL-6) expression in RAW 264.7 macrophages compared to PGE3 [112], suggesting that omega-6 fatty acids stimulate an inflammatory response. More importantly, they show that replacing omega-6 with omega-3 in the lipid membrane decreases the cell’s response to inflammatory stimuli [112]. Another enzyme that metabolizes omega-6 and omega-3 into proinflammatory and anti-inflammatory eicosanoids is 12/15-lipoxygenase (12/15-LO) [113]. In 12/15-LO knockout mice fed a high-fat diet, they were demonstrated to be protected from insulin resistance, hepatic lipid accumulation, and inflammation compared with controls fed the same diet [113]. In human association studies, omega-6 polyunsaturated fatty acids (PUFAs) were positively correlated with hyperinsulinemia [114] and MASLD [115]. Overeating saturated fatty acids promotes the accumulation of hepatic triglycerides and serum ceramides, while PUFAs in the diet reduce both [116]. Individuals who are overweight and eat meals high in saturated fatty acids are more likely to become insulin resistant and have escalated DNL and elevated plasma ceramides compared with those who eat unsaturated fatty acids [117]. Mechanistically, excess saturated fatty acids induce apoptosis, expression of proinflammatory cytokines, and impair insulin signaling [118,119]. These studies demonstrate that an imbalance in saturated and unsaturated fatty acids can lead to lipid accumulation and inflammation in MASLD that progresses to an insulin-resistant state.

### Mechanisms causing hepatic fat accumulation and insulin resistance

#### Hepatic *de novo* lipogenesis and insulin resistance

**Figure 5:**
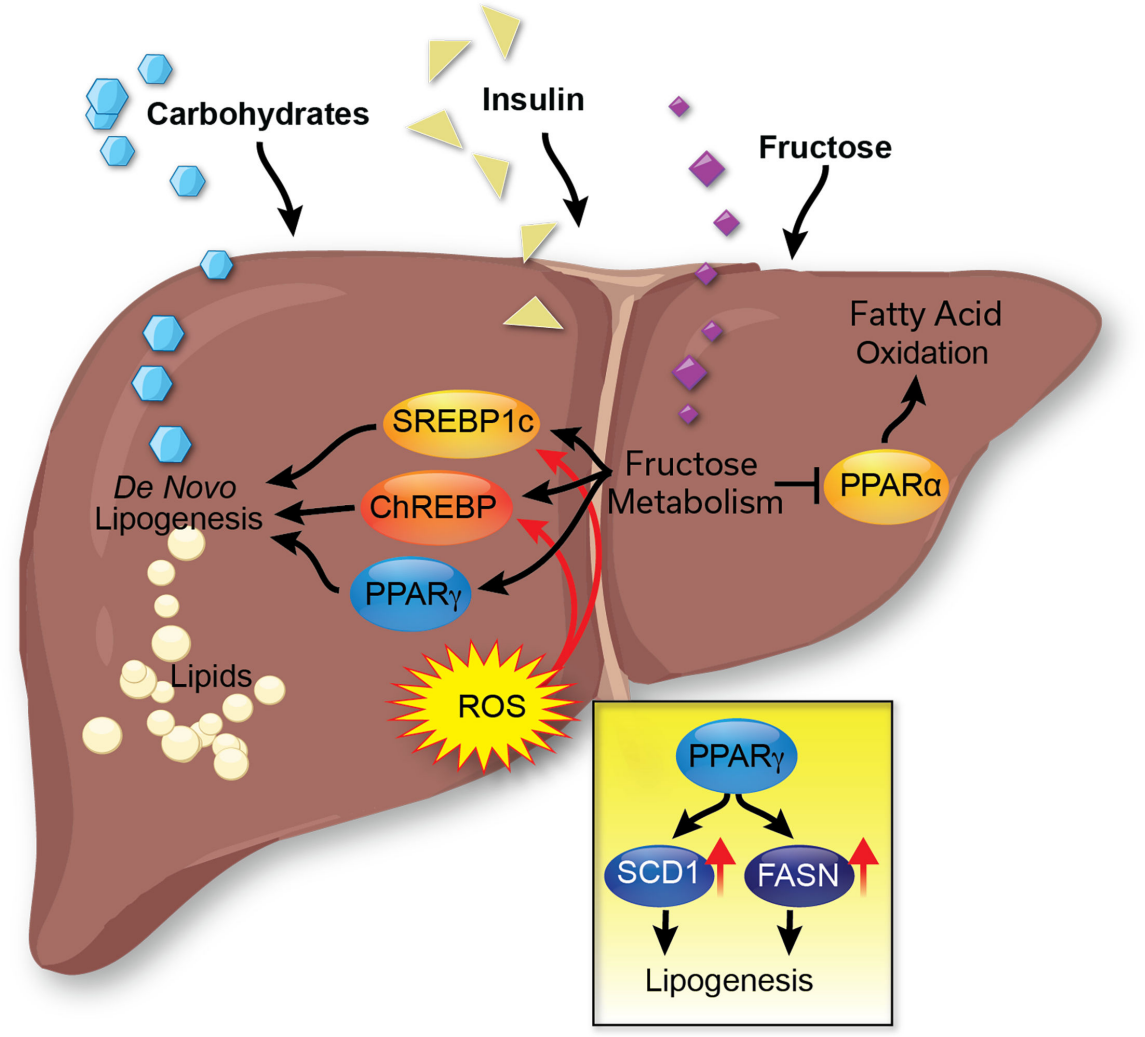
The regulation of hepatic *de novo* lipogenesis. In the liver, *de novo* lipogenesis produces lipids (primarily stored as triglycerides) from carbohydrates and glucose. This process is mainly regulated by the transcription factors SREBP1c and ChREBP. To activate lipogenesis, these proteins can be induced by insulin, reactive oxygen species (ROS), and fructose metabolism.

Hepatic *de novo* lipogenesis is an indispensable process in the liver to meet nutrient demand by producing fatty acids from excess carbohydrates and glucose (Figure 5) [120]. During nutrient overload, the process begins with glucose entering the cell and then escalating the glycolysis cycle, producing pyruvate, which is later made into acetyl-CoA, the starting product of DNL. The acetyl-CoA is then converted into malonyl-CoA by acetyl-CoA carboxylase (ACC). Fatty acid synthase (FASN) generates the saturated fatty acid palmitate (16:0) from malonyl-CoA. Stearoyl-CoA desaturase 1 (SCD1) creates a monoacylglycerol (18:1) from palmitate to begin producing triglycerides. Monoacylglycerol acyltransferase (MGAT) and diacylglycerol acyltransferase (DGAT) attach two more fatty acids, creating a triglyceride that has a glycerol head and three fatty acids attached, which is the main form of fat storage in the liver. Palmitate can also be unsaturated or elongated before the formation of triglycerides. The overactivation of the DNL pathway can manifest in several diseases, including MASLD [121], which occurs with hepatic insulin resistance [75].

In the normal healthy liver, insulin promotes hepatic DNL to produce fatty acids from carbohydrates for energy storage postprandially. Hepatic insulin increases DNL by activating the transcription factor sterol regulatory element binding protein-1c (SREBP1c) by increasing its gene transcription and protein maturation [122]. The mature SREBP1c then increases the transcription of ACC, FASN, and SCD1, leading to an up-regulation of DNL [123]. During hepatic insulin resistance, it has been hypothesized that insulin activates SREBP1c, and due to hyperinsulinemia, this pathway is overactivated, leading to excessive DNL [124]. In a study of over 65,000 patients, the concentration of fatty acids known to be produced by DNL was positively associated with the incidence of T2DM [125]. In patients with MASLD, elevated glucose and insulin-stimulated hepatic DNL lead to an increase in intrahepatic triglycerides [75]. Hepatic DNL is negatively associated with liver and peripheral insulin sensitivity [75]. Hence, fat accumulation in the liver leads to hepatic insulin resistance. After a prolonged period of fat accumulation in the liver, it manifests as oxidative stress, causing hepatocyte death and liver injury. These aspects are discussed further in the next sections.

#### Oxidative stress and liver lipogenesis

Patients with MASLD have a hepatic elevation of reactive oxygen species (ROS) [126]. There is a cross-talk between ROS and lipogenesis in the liver, which can activate each other (Figure 5). The excessive accumulation of lipids in hepatocytes from either diet, DNL, or the release from adipose tissue causes increased β-oxidation and oxidative phosphorylation, leading to mitochondrial dysfunction and increased cellular ROS [127]. The buildup of cellular ROS affects intracellular insulin and lipogenesis signaling pathways [128]. ROS regulates lipogenesis by activating SREBP1c, leading to an up-regulation of lipogenesis proteins, as discussed in [129]. ROS also induces the expression of carbohydrate response element-binding protein (ChREBP) through hepatocyte nuclear factor 4α (HNF4α), activating lipogenesis via FASN and SCD1 [130]. An increase in cellular hydrogen peroxide (H_2_O_2_) lowers the ability of phosphatases to dephosphorylate their targets, which either increases or decreases kinase signaling depending on the phosphorylation site. ROS also activates c-Jun N-terminal kinase (JNK), which inhibits insulin receptor signaling [131] and reduces glucose tolerance [132], suggesting that ROS suppresses insulin sensitivity. ROS also stimulates the transcription factor, NF-κB (Nuclear Factor-κB) and inflammatory pathways [133], which together could explain the increased ROS observed in people with obesity and increased adiposity and inflammation.

Bilirubin is a strong antioxidant that suppresses ROS and reduces oxidative stress levels [60,134–136]. Plasma bilirubin levels are lower in people with obesity [106]. A bilirubin deficiency results in oxidative stress and inflammation [137]. A CRISPR knockout of biliverin reductase A (BVRA), which is the enzyme that generates bilirubin [61,136,138] in hepatocytes, caused significantly increased levels of ROS generation and decreased levels of superoxide dismutase mRNA expression [139]. The study observed that hepatocytes with reduced BVRA had a reduction in mitochondrial number, decreased expression of markers of mitochondrial biogenesis, uncoupling, oxidation, and fusion, which paralleled reduced mitochondrial oxygen consumption. These observations were also made in mice with a liver-specific deletion of BVRA, which, when fed a high-fat diet, had significantly higher hepatic lipid accumulation and oxidative stress [140]. Mice with an adipose-specific BVRA knockout fed a high-fat diet also had increased adiposity in their adipose tissue compared with flox control mice and decreased mitochondrial content and elevated ROS biomarkers [141].

Mice with hyperbilirubinemia due to the human UGT1A1*28 Gilbert’s syndrome polymorphism fed a high-fat diet had significantly lesser hepatic fat accumulation, reduced plasma cholesterol, insulin, and glucose sensitivity [142]. Bilirubin nanoparticles treatment in mice with obesity and MASLD observed similar findings to those in humanized mice with Gilbert’s polymorphism; the bilirubin nanoparticles significantly reduced liver fat content, adiposity, and blood glucose in a murine MASLD model [54,56,62]. Overall, regulating plasma bilirubin levels or exogenous delivery of bilirubin nanoparticles may be useful for reducing hepatic ROS, adiposity, inflammation, and insulin resistance associated with MASLD.

#### Hepatic insulin clearance and fat accumulation

Two genes, insulin-degrading enzyme (IDE) and CEACAM1, have been identified as regulators of insulin clearance from the blood (discussed further in [1]). Both have been demonstrated to participate in insulin receptor-dependent endocytosis [143,144] and insulin-degrading processes [145,146], which ultimately mediate circulating insulin levels. IDE may have an additional role in insulin receptor recycling to the membrane [147]. Mice with a liver-specific IDE knockout, fed a high-fat diet, developed worsened hepatic insulin resistance and glucose intolerance [148], but this did not affect insulin clearance compared with controls fed the same diet [149]. IDE improves hepatic insulin resistance by increasing glucose transporter 1 (GLUT1) expression [148]. IDE has a well-established role as an insulin protease and in regulating insulin turnover (reviewed further in [150]). For these reasons, we will expand our discussion on CEACAM1 in liver pathology of insulin resistance and MASLD, as it is an emerging significant player in disease progression.

CEACAM1 is a transmembrane protein that can promote hepatocyte insulin degradation and inhibit FASN [147]. The process starts with insulin binding to its receptor and the formation of an endosome [147]. CEACAM1 binds to the activated insulin receptor and enters the hepatocyte cytosol through endocytosis, where it can interact with FASN and inhibit its activity [151]. This process can counterbalance the surge in insulin-induced fatty acid synthesis when a high concentration of freshly released insulin reaches the liver after a meal [152]. It also indicates that CEACAM1 has inhibitory effects on hepatic fat accumulation. The CEACAM1-dependent endocytosis of insulin-bound insulin receptors facilitates the degradation of insulin and maintains the normal insulin half-life in the circulation, thereby preventing hyperinsulinemia and systemic insulin resistance. Numerous studies have demonstrated that reduced CEACAM1 is involved in the development of MASLD and MASH. Mice with a global *Ceacam1* null mutation fed a high-fat diet to induce obesity exhibited worsened liver steatosis, increased hepatic inflammation, and stimulated fibrosis in the knockout, but no fibrosis was observed in control mice on the same diet [153]. A CEACAM1 deletion in human LX2 HSCs caused significant growth and proliferation and activation of collagen-producing genes [154]. This study also showed that mice with an HSC-specific CEACAM1 knockout displayed hepatic inflammation and fibrosis but without insulin resistance or hepatic steatosis. In immune cells, CEACAM1 has been reported to inhibit T-cell activation and inflammation [155], which postulates the idea that hepatic inflammation observed in CEACAM1-mutant mice may have resulted from the loss of CEACAM1’s anti-inflammatory effects. CEACAM1 protects the liver from MASLD and MASH by maintaining normal hepatic insulin clearance, reducing excessive FASN-induced hepatic fat accumulation and inflammation.

Mice fed a high-fat diet for three weeks exhibited significantly reduced hepatic CEACAM1 expression by more than 50 percent, accompanied by observations of hyperinsulinemia, insulin resistance, and elevated liver triacylglycerol levels [156]. Additionally, the same investigation demonstrated that the rescue of CEACAM1 expression via adenovirus reversed high-fat-induced insulin resistance and liver steatosis in mice [157]. In humans, decreased liver CEACAM1 expression is significantly more prevalent in individuals with MASLD and severe obesity, regardless of their diabetic status [158]. These findings suggest that a deeper understanding of CEACAM1 could reveal its importance in preventing hepatic insulin resistance associated with MASLD development and progression and could be a potential area of investigation for therapeutics targeting MASLD.

#### Fructose metabolism and fat accumulation in the liver

A diet high in fructose can induce hepatic steatosis without weight gain [159]. The dietary intake of foods high in fructose is associated with an increased risk of MASLD. The risk of MASLD development is up to three times higher in individuals who frequently consume foods high in fructose compared with those who abstain from consuming such foods [160]. Fructose is almost completely metabolized in the liver (approximately 70% of oral fructose consumed), beginning with ketohexokinase (KHK) [161,162]. A key difference between glucose and fructose metabolism is their rate of regulation. Glycolysis has a rate limit determined by phosphofructokinase (PFK) activity, which can be inhibited by high ATP and citrate concentrations [163]. This rate-limiting effect ensures that glucose metabolism occurs only when energy production is needed. However, rate-limiting mechanisms are not observed in fructose metabolism. Therefore, with excessive intake of fructose, there is a constant and unlimited metabolism of fructose in the liver, which leads to an elevation of substrates for lipogenesis. The catabolism of fructose in the liver leads to the activation of SREBP1c [164] and ChREBP [165], which increases the expression of lipogenic enzymes independently of the effects of insulin (Figure 3). From a clinical standpoint, KHK inhibition represents potential as a therapeutic approach. For example, PF-06835919, a KHK inhibitor in phase 2 a study, has been shown to correct metabolic abnormalities and reduce hepatic steatosis, with no serious adverse events observed [166].

Glycolysis metabolites, such as glucose-6-phosphate and fructose-2,6-bisphosphate, activate ChREBP [167], suggesting that glucose metabolism contributes to hepatic lipid accumulation. However, compared with fructose metabolism, the lipid-accumulating effect of glucose metabolism is rate-limited and more tightly regulated. Fructose can also increase intracellular ROS in hepatocytes, indirectly leading to lipogenesis [168]. In addition to stimulating lipogenesis, fructose decreases fatty acid oxidation by inhibiting CPT1A [169] and PPARα [170], indicating its inhibitory effects on hepatic fat burning.

#### PPARγ and liver fat accumulation

PPARα and PPARγ are nuclear receptor ligand-activated transcription factors that bind to gene promoters to regulate the transcription of their target genes and are important for regulating hepatic lipids (Figures 3 and 5). PPARγ drives the expression of the lipogenic enzymes FASN and SCD1 and lipid uptake genes fatty acid transport protein (FATP) and CD36 [171]. The hepatic expression of PPARγ is elevated in patients with obesity and MASLD [172]. The loss of PPARγ in hepatocytes protects against diet-induced hepatic steatosis in animal models [173]. The role of PPARγ may switch to protective during the later stages of liver disease as the overexpression of PPARγ in hepatic stellate cells (HSCs) protects against fibrosis [174]. PPARγ agonist therapy has hepatoprotective effects against fibrosis primarily through stimulating the adipose tissue to take up the free fatty acids, which lowers the burden on the liver and releases adiponectin, as discussed in the adipose section later.

The interesting and confusing paradox with adiponectin is that it is almost exclusively expressed in adipocytes; however, a CRISPR knockout of its gene *ADIPOQ* in human LX2 HSCs and their activation with transforming growth factor-β (TGFβ) stimulated fibrotic pathways substantially higher than scramble CRISPR control cells treated with the same [174]. In the same study, it was shown that the loss of *AdipoQ* expression specifically in murine HSCs also demonstrated significantly worsened liver fibrosis. This conundrum may be explained by HSCs typically storing vitamin A and fatty acids, which hepatocytes do not, and activated HSCs proliferate and secrete their lipids along with collagen, causing fibrosis. PPARγ in HSCs is likely inhibiting the loss of fatty acids and HSC activation.

Thiazolidinediones (TZDs) are a class of PPARγ agonists that have long been used as insulin sensitizers and treatments for metabolic diseases, including MASLD [175]. TZDs increase insulin sensitivity through promoting adipocyte differentiation from mesenchymal stem cells and enhancing lipogenesis in visceral adipose tissues [176]. Common TZDs include rosiglitazone and pioglitazone, with the latter emerging as the preferred agent due to its demonstrated efficacy in patients with MASLD and MASH [175,177]. While rosiglitazone has been associated with an increased risk of myocardial infarction, pioglitazone presents comparatively fewer cardiovascular concerns [175]. However, pioglitazone still increases the risks of fracture and bladder cancer [175,178,179], which limit its clinical use and growth on the market. Although pioglitazone shows potential as a therapy for MASLD-induced liver fibrosis by ameliorating metabolic disorders and liver steatosis, the efficacy remains inconclusive. For example, a mouse study has shown that 16 weeks of pioglitazone treatment at 10  mg/kg/d did not affect high-fat feeding‐induced liver fibrosis signatures [180]. Further studies, especially in humans, are needed to establish pioglitazone’s possible protective role in fibrosis prevention and therapy.

### Fatty liver progression with hepatic fibrosis

**Figure 6:**
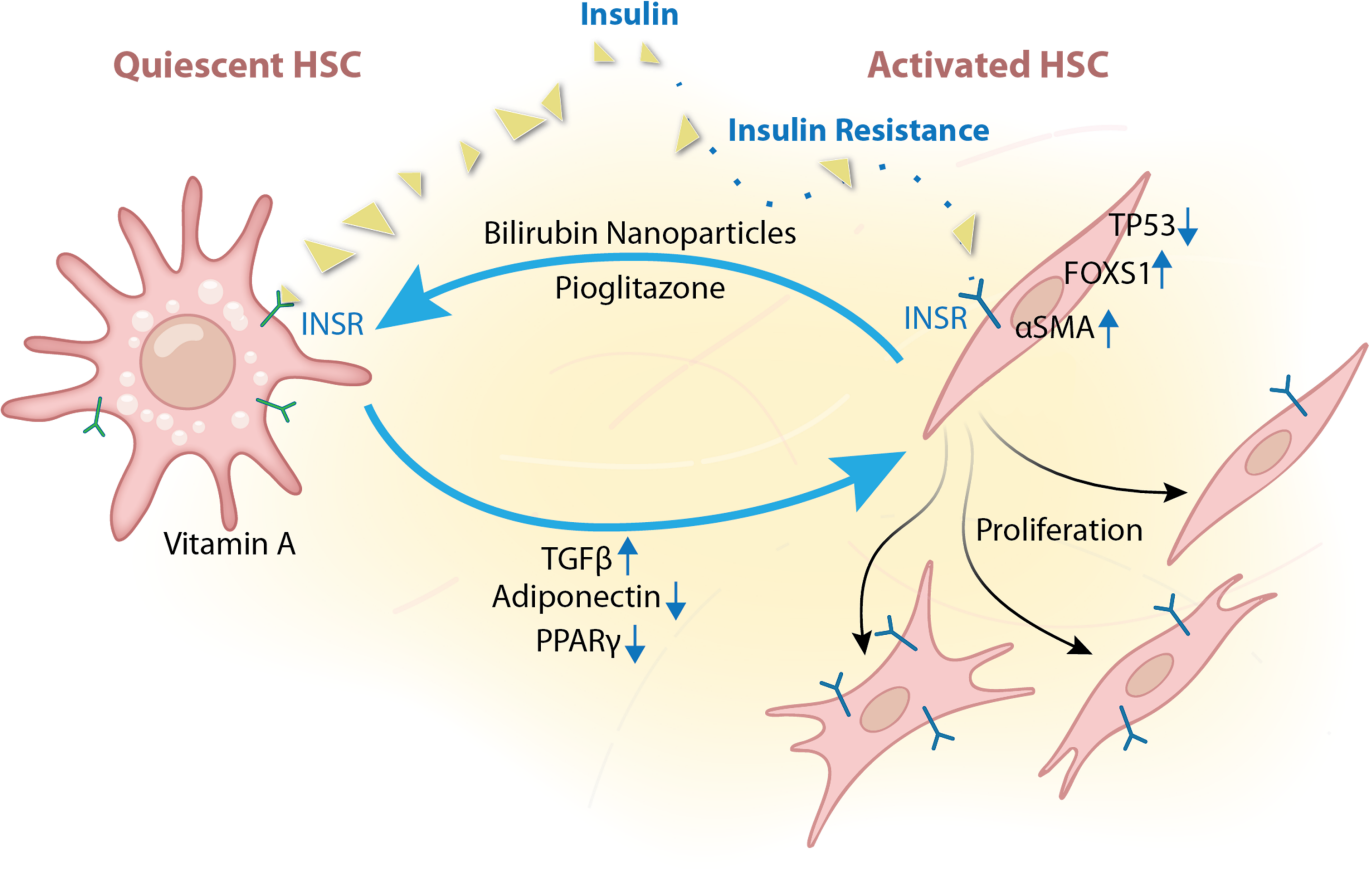
Hepatic stellate cell (HSC) activation in liver fibrosis. Quiescent HSCs (qHSCs) can be activated by TGFβ stimulation in liver fibrosis. Reduced adiponectin and PPARγ activities have also been shown to activate HSCs. Activated HSCs (aHSCs) are less sensitive to insulin and have increased *FOXS1* (HSC activation marker) expression. Insulin resistance also decreases *TP53* expression and increases HSC proliferation and αsma (HSC activation marker) expression. TGFβ, transforming growth factor-β.

Once the liver has prolonged fat accumulation, it causes hepatocyte ballooning and oxidative stress damage that causes hepatocytes to release transforming growth factor-β (TGFβ) [1,60]. Elevated levels of TGFβ activate signaling cascades that induce insulin resistance [181,182]. Most notably, TGFβ also activates HSCs to proliferate and secrete collagen, leading to fibrosis (Figure 6) [1,60,183]. TGFβ, through Smad3 (small mothers against decapentaplegic 3), induces increased body weight, glucose intolerance, hepatic lipid accumulation, and reduced insulin sensitivity in mice fed a high-fat diet [182]. Mice fed a diet high in fat with liver-specific TGFβ deletion compared with wildtype controls had significantly improved glucose tolerance and insulin sensitivity, reduced liver weights, decreased hepatic triglyceride levels, and less liver fibrosis than controls [184]. In high-fat diet-fed rats, TGFβ mRNA was increased along with phosphorylation of ERK1/2 and AKT signaling in the epididymal WAT [185]. In humans, TGFβ is positively correlated with obesity and insulin resistance [184]. Inhibition of TGFβ downstream signaling pathways may be a potential therapeutic for TGFβ-induced insulin resistance. The insulin receptor governs TGFβ-induced HSC activation, and CRISPR-induced insulin resistance in human HSCs activates liver fibrotic pathways [186]. The transcription factor Forkhead Box 1 (FOXS1) is an emerging biomarker in liver fibrosis [187], and it could be that it is essential for insulin resistance to initiate fibrotic pathways, as CRISPR FOXS1 knockout in human LX2 HSCs increased the kinase activity of the insulin receptor [187].

Interestingly, Shinn et al. demonstrated that bilirubin nanoparticles counteract the lipid-reducing effects of HSC activation, a critical process in the initiation of liver fibrosis [188]. Although they did not investigate whether PPARα mediated the anti-fibrotic activity of the bilirubin nanoparticles, they did establish that these nanoparticles significantly decreased liver fat content, reduced levels of fibrosis biomarkers such as αSMA and others, and exhibited anti-fibrotic properties. This finding was surprising because humans with cirrhosis have end-stage fibrosis and usually very high bilirubin levels [183].

A conundrum has been observed, where humans with cirrhosis have significantly higher kinase activity of the HSC insulin receptor, but not in hepatocytes [183]. This finding was validated in a carbon tetrachloride (CCl_4_)-induced murine model of liver fibrosis [183]. However, this was not observed in liver fibrosis from a high-fat diet with fructose, which had reduced insulin receptor kinase activity [183]. In considering these differences, body weight may be a significant contributor to the insulin receptor responsiveness in patients with liver fibrosis or cirrhosis. Patients with cirrhosis typically have reduced muscle mass that leads to sarcopenia and increased visceral obesity, crowning the condition of ‘sarcopenic obesity’ [189]. For these reasons, herein, we will discuss the function of adipose tissue depots and their impact on liver dysfunction in MASLD.

## Part 2: Adipose-specific metabolic signaling

### Molecular actions of adipose fat accumulation and insulin resistance

#### Adipose tissue lipogenesis and insulin resistance

There are generally two mechanisms for how adipose tissue accumulates lipids. One mechanism is the uptake of VLDL particles secreted from the liver for storage in adipose tissue. The second mechanism is lipogenesis, which occurs directly in adipose tissue after insulin stimulation [190]. This process begins with glucose uptake, primarily through the glucose transporter type 4 (GLUT4) in muscle and adipose tissues [1]. Under basal conditions, GLUT4 resides in the trans-Golgi network until insulin stimulates the cell, which induces its translocation to the cell membrane, where it uptakes extracellular glucose [191]. Once the glucose enters the cell, it is metabolized into pyruvate through glycolysis [190]. Pyruvate is needed to produce acetyl-CoA, which is essential for delivering an acetyl group into the Krebs cycle for ATP synthesis or the production of lipids.

The subcellular localization of acetyl-CoA differs between fasted and fed states. In the fasted state, acetyl-CoA is localized in the mitochondria to perform survival functions, including ATP generation [192]. In the fed state, acetyl-CoA is distributed throughout the cytosol and nucleus for lipid synthesis [192]. Acetyl-CoA is converted to malonyl-CoA by ACC, and this is used as a substrate by FASN to add two carbon chains to a growing fatty acid chain that eventually forms palmitate [193]. Palmitate, a 16-carbon saturated fatty acid, is desaturated by SCD1 to form monounsaturated fatty acids to be esterified to glycerol to generate triglycerides and directly stored in adipocytes or the liver. SREBP1 and PPARγ regulate these lipogenic enzymes (ACC, FASN, and SCD1) [190]. Also, insulin increases glucose uptake in adipocytes, which can be used for lipogenesis. Insulin stimulates mTOR activation of SREBP1c and indirectly activates ChREBP, which both promote *de novo* lipogenesis [194]. Insulin also promotes the process of glyceroneogenesis to produce glycerol for triglyceride synthesis [194].

Adipose tissue insulin resistance is generally characterized by an increased release of free fatty acids into the bloodstream despite hyperinsulinemia [195]. A consequence of increased fatty acid release into circulation is ectopic fat accumulation in internal organs such as the stomach, kidneys, and liver. Expansion of subcutaneous and visceral adipose depots is strongly correlated with the development of MASLD [196,197]. Insulin resistance in adipose tissue is associated with impaired lipogenesis and adipocyte hypertrophy [198]. The occurrence of insulin resistance in adipose tissue initiates inflammation [199].

**Figure 7:**
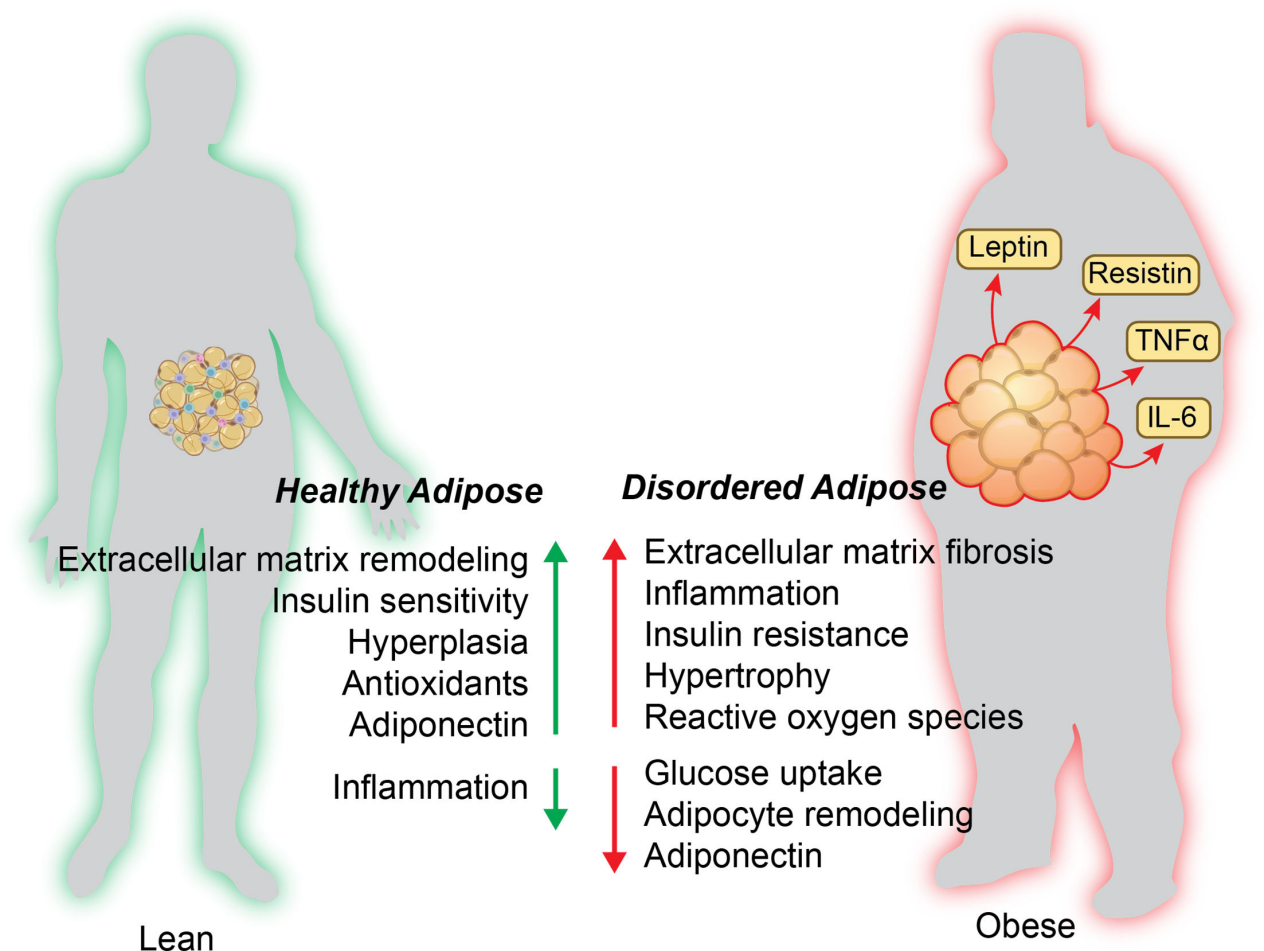
Characteristics of adipose tissue from lean and obese individuals. Healthy adipose tissue is typically characterized by extracellular matrix remodeling, insulin sensitivity, and hyperplasia. In contrast, obese individuals have disordered adipose tissue characterized by hypertrophy, fibrosis, insulin resistance, and reduced adipocyte remodeling.

In general, obesity and insulin resistance are associated with increased proinflammatory cytokines from the adipose (Figure 7), including leptin [200], IL-6, TNFα, and resistin [201]. The state of chronic inflammation in the adipose tissue further confounds the ability of the tissue to be insulin sensitive [202]. One possible mechanism is the recruitment and activation of macrophages in adipose tissue. The increased free fatty acid release activates CD11c(+) myeloid proinflammatory cells in adipose tissue [203]. The free fatty acids regulate macrophage activation through the toll-like receptor 2 (TLR2) and TLR4 and promote the phosphorylation of JNK, which increases the expression of pro-inflammatory genes like IL-1β and tumor necrosis factor α (TNFα) [203]. TLR2 and TLR4 knockout mice fed an HFD do not exhibit increased levels of JNK phosphorylation and have reduced levels of adipose tissue inflammatory markers [203,204]. Altered adipokine secretion has also been implicated as a consequence of insulin resistance in adipose tissue [201]. The adipose tissue secretes less anti-inflammatory adipokines in response to obesity, such as adiponectin, omentin-1, and secreted frizzled-related protein 5 [201].

#### Brown adipose tissue in fat metabolism

In WAT, the primary function is to store energy in the form of triglycerides. In contrast, brown adipose tissue (BAT) has a higher mitochondrial count and functions by primarily burning triglycerides through mitochondrial uncoupled respiration [205]. BAT is characterized by its location on the body and high expression of thermogenic genes, most notably UCP1. It is vital in generating heat by uncoupling the mitochondrial proton gradient during mitochondrial respiration [206]. UCP1 acts as a long-chain fatty acid anion/H^+^ symporter, which is how UCP1 transports H^+^ to change the mitochondrial H^+^ gradient to produce heat [207]. Other genes that have increased expression in BAT include PGC1α (*PPARGC1A*), PR domain containing 16 (*PRDM16*), and β3 adrenergic receptor (*ADRB3*), which are important regulators for BAT differentiation and thermogenesis [208,209]. Studies in healthy adults showed a 15-fold increase in glucose uptake in BAT when acutely exposed to the cold [209], which is utilized to regulate body temperature and not for energy storage, as seen in the WAT. In addition to cold exposure, BAT activation can also occur through insulin stimulation [210], which may be perturbed in states of insulin resistance.

In general, obese individuals typically have less BAT compared with their lean counterparts [211]. The BAT size positively correlates with humans' insulin sensitivity [211]. In rodent models, the removal of BAT induced significant weight gain, inflammation, and insulin resistance [212], indicating the important role of BAT in metabolic health. Studies in the adipose tissue of obese mice also show that BAT has a much lower number of macrophages and inflammatory responses compared with WAT [211]. BAT activation has been explored for its use in alleviating obesity-associated comorbidities like insulin resistance and impaired glucose homeostasis, as well as MASLD. In a study of eight patients with diabetes, cold acclimation to induce BAT improved insulin sensitivity by 43% [213]. Future studies are needed to determine the therapeutic potential of targeting BAT activation for metabolic diseases.

#### PPARs and regulation of adipose tissue lipid accumulation

The PPAR family consists of three isoforms: PPARα, PPARγ, and PPARδ. PPARα and PPARδ mediate β-oxidation and fat utilization, with PPARδ being highest expressed in muscle and PPARα predominantly in the liver. PPARγ is the highest expressed in adipose tissues and controls several key pathways of adipogenesis and lipogenesis. PPARγ exists in two sub-isoforms, where PPARγ1 is ubiquitously expressed throughout many tissues, and PPARγ2, an alternatively spliced isoform with an additional 30 amino acids on its N-terminus [178], is primarily localized in adipose tissue.

The expression of *PPARG* is significantly higher in patients with obesity and positively correlated with BMI, waist circumference, and weight-hip ratio [214]. The expression of PPARγ2 RNA was markedly higher in the subcutaneous adipose tissue of female and male obese humans compared with lean controls and was reduced with a hypocaloric diet [215]. The expression of PPARγ was also higher in the visceral adipose tissue of obese humans compared with lean, especially in people who were sedentary [216]. After bariatric surgery, patients who lose >33% of their body weight have significantly lower PPARγ expression in the visceral and subcutaneous adipose depots than those who had ≤33% [217]. The presence of PPARγ is required for adipogenesis *in vivo* and *in vitro* [218], and the heterozygous knockout of PPARγ in mice protected against HFD-induced weight gain and fat mass accumulation with a ~70% reduction in WAT mass [218]. Studies using adipose-specific PPARγ knockout (PPARγ^AKO^) mice found reduced WAT and BAT mass after standard chow and a high-fat diet [219]. Jones et al. demonstrated that the PPARγ^AKO^ mice had reduced fat mass and improved insulin sensitivity compared with control mice, when both groups were fed a diet high in fat to induce obesity [220]. The knockout studies validate PPARγ’s function in adipogenesis and lipid accumulation in the adipose.

In homeostatic conditions, stimulating adipocytes with insulin increases PPARγ activity, promotes lipogenesis, and suppresses lipolysis. Increased PPARγ activity is associated with increased insulin sensitivity, as displayed in patients treated with TZD PPARγ agonist rosiglitazone or pioglitazone therapies [221]. People with obesity who are nondiabetic and treated with pioglitazone showed a reduction in the size of adipocytes and an increase in the proportion of smaller adipocytes in the total population [222,223]. This was associated with increased insulin sensitivity and redistribution of adipose depots from visceral to subcutaneous depots, a known phenomenon of pioglitazone treatment [222]. TZDs, such as rosiglitazone and troglitazone, are thought to function via adipose tissue to reduce blood glucose and insulin levels [224]. PPARγ also regulates insulin sensitivity by activating anti-inflammatory pathways [225]. The activation of PPARγ reduces circulating resistin [226] and monocyte-derived TNFα, IL-6, and IL-1β [227].

Ma et al. demonstrated by chromatin immunoprecipitation sequencing (ChIP-seq) studies that PPARγ binds and regulates the promoters of *FASN*, *ACC*, and *SCD1* to induce *de novo* lipogenesis [228]. It is known that several post-translational modifications affect PARγ activity, some of which are linked to obesity and metabolic syndrome. The serine 273 (Ser273) phosphorylation of PPARγ is regulated by cyclin-dependent kinase 5 (CDK5) [205] and extracellular signal-regulated kinase (ERK) and is higher in mouse models of diet-induced obesity [229]. The phosphorylation at Ser273 controls which pathways PPARγ activates, as it does not affect the adipogenic ability of PPARγ, but inhibits its insulin-sensitizing capabilities and the expression of adiponectin [229]. Ser273 has a therapeutic interest in targeting PPARγ to improve insulin sensitivity without the weight gain observed in mice treated with rosiglitazone or pioglitazone [230,231]. Another important phosphorylation site of PPARγ is located at Serine 112 (Ser112), which regulates PPARγ activity depending on which kinase is responsible for the phosphorylation; MAPKs decrease PPARγ activity [232], while CDK7 and CDK9 increase PPARγ activity [205,233]. The dephosphorylation of the Ser112 site in PPARγ is controlled by protein phosphatase 5 (PP5), which has been demonstrated to directly bind to the PPARγ-heat shock protein 90 (HSP90) complex [234]. The mutation of serine 112 to alanine (S112A) in adipocytes increased their ability to differentiate in the presence of a PPARγ ligand [232]. Another study confirmed that the mutation of Ser112 to alanine (S112A) increases the activity of PPARγ and promotes bone marrow stromal cells to differentiate into adipocytes instead of osteoblasts [235]. Future studies are needed to investigate the regulation of PPARγ phosphorylation sites and their roles in metabolic diseases.

While PPARα is less expressed in the adipose tissue than PPARγ, it still has a regulatory function. The loss of PPARα specifically in the adipose tissue (PPARα^AKO^) led to increased adiposity in the inguinal WAT (iWAT) and BAT in male mice fed an HFD [236]. The knockout studies observed that PPARα in the adipose tissue inhibits lipogenesis and inflammation in diet-induced obesity in a sexually dimorphic way. Female adipose-specific knockout animals did not affect adiposity. However, the male PPARα^AKO^ mice had significantly higher precursor and mature SREBP1 protein, leading to an increased expression of FASN and SCD1 in the iWAT [236]. The male PPARα^AKO^ mice also significantly increased the mRNA expression of nitric oxide synthase 2 (*Nos2*)*,* an M1 macrophage marker, indicating a switch from the anti-inflammatory M2 to the pro-inflammatory M1 [236]. In obese diabetic male mice, the treatment of WY-14,643, a PPARα agonist, reduced adipocyte size, WAT mass, and adipose inflammation [237]. The bilirubin treatments in obese mice also significantly reduced adipocyte size in WAT but not BAT and increased the mitochondrial content and induced thermogenic genes *Ucp1* and *Adrb3* in WAT, reducing adiposity [54]. Further experiments are needed to determine whether PPARα is essential for bilirubin’s effects in adipose tissue.

Anti-metabolic disorder therapies targeting PPARα and PPARγ include fibrates, TZDs, and dual agonists. Fibrates, which are PPARα agonists, such as fenofibrate, have been widely used to treat hyperlipidemia and have also been shown to reduce hepatic steatosis and MASLD [238]. Other fibrates, including bezafibrate [239], ciprofibrate [240], clofibrate [241], and gemfibrozil [242], activate PPARα and exert anti-dyslipidemic effects. While fibrates have long been adopted for treating metabolic disorders, side effects like liver dysfunction and an increase in creatinine levels have been reported [243]. New fibrates are being developed to provide safer alternatives. For example, pemafibrate is an emerging PPARα agonist that has the potential to reduce adverse side effects while enhancing therapeutic effects [243]. However, more testing in humans is needed to better understand its usefulness.

PPARγ agonist, such as TZDs, has also been extensively used to treat metabolic disorders due to their potent insulin-sensitizing properties. However, several TZDs have been withdrawn or limited by the FDA due to concerns over hepatic toxicity (e.g., troglitazone) or cardiovascular risks (e.g., rosiglitazone) [244]. Some compounds, such as aleglitazar and saroglitazar, act as dual PPARα and PPARγ agonists and have been demonstrated to improve metabolic dysfunction [244–246]. Although these dual agonists have shown benefits against insulin resistance and MASLD, their long-term consequences and side effects are incompletely understood. Further studies are needed to elucidate how dual activation of PPARα and PPARγ contributes to the amelioration of metabolic dysfunction.

#### Oxidative stress in adipose tissue and lipogenesis

An important regulator of adipocyte ROS concentration is NADPH oxidase 4 (NOX4), which acts as an oxygen sensor that produces superoxide anions (O^-^) and H_2_O_2_ [247]. NOX4, one of four NOX isoforms, is the most expressed isoform in adipose tissue and is constitutively active, producing oxidants [247]. In healthy WAT, H_2_O_2_ produced from NOX4 activation is essential for NADPH production, the incorporation of glucose into triglycerides, and the activation of pyruvate dehydrogenase [248]. These processes increase lipogenesis and healthy adipose tissue remodeling [248]. H_2_O_2_ activates MAP kinase phosphatase (MPK-1), which is essential for ERK1/2 inhibition by NOX4 [249]. This ERK1/2 inhibition is important because it inhibits the insulin receptor from activating insulin receptor substrate 1 (IRS-1), making NOX4 a negative regulator of adipocyte proliferation and a positive regulator of differentiation [249].

NOX4 expression in adipocytes is increased in response to excessive glucose [250] and has a role in the differentiation process as demonstrated in 3T3-L1 adipocytes [251]. NOX4 and the NOX family are essential in adipose expansion, adipokine release, and extracellular matrix remodeling [252]. NOX4 signaling is dysregulated in obesity, contributing to insulin resistance, extracellular matrix fibrosis, and hypertrophy observed in WAT of people with obesity [252]. The mechanism for how this occurs is unknown, but association studies in humans indicated that NOX4 mutations may be related to the development of obesity [253]. Obesity causes adipocyte hypertrophy, inflammation, oxidative stress, and insulin resistance in adipose tissues, which are associated with adipose dysfunction (Figure 7).

### Interorgan signaling between adipose and liver tissue in insulin resistance

#### Cytokines and adipokines in inflammation in the adipose–liver axis

Proinflammatory cytokines and adipokines are signaling molecules that induce or worsen an insulin-resistant state by inhibiting insulin signaling in numerous tissues, especially in the adipose and liver (Figure 7) [254]. A major proinflammatory cytokine involved in the impairment of insulin signaling is TNFα. Uysal et al. demonstrated that TNFα null mice (TNFα^-/-^) fed a diet high in fat to induce obesity were protected against hyperinsulinemia and had a 26% decrease in serum triglyceride levels [255]. In elderly male and female patients with T2DM, there is a positive correlation between plasma TNFα levels and HbA1c levels over two years [256]. Patients with a TNFα polymorphism in its gene’s promoter (−238 G/A) had a higher occurrence of insulin resistance and impaired glucose tolerance [257]. On the molecular level, TNFα increases the phosphorylation of IRS-1 and decreases the phosphorylation of AKT substrate 160 (AS160) [258], indicating impairment of insulin signaling that reduces glucose uptake. Other proinflammatory cytokines implicated in the impairment of insulin signaling leading to insulin resistance in the liver and adipose include IL-1β and IL-6 [259].

Adipokines such as leptin and adiponectin signal to the liver, regulating inflammation, lipid metabolism, and insulin sensitivity [260–267]. Leptin and adiponectin have opposing effects: Increased leptin is associated with insulin resistance [268–270] and an increase in proinflammatory cytokines [271–277], while increased adiponectin is associated with insulin sensitivity [262] and anti-inflammatory effects [278]. In the liver of adiponectin knockout mice [279] and primary rat adipocytes [280], adiponectin supplementation activated AMPK and decreased TNFα mRNA, indicating that it activates energy-dependent pathways and reduces inflammation.

##### TLR and ligands

Two TLRs recognize lipopolysaccharides and saturated fatty acids in the liver and adipose tissues [281,282], including TLR2 [283,284] and TLR4 [285,286]. TLR2 binds a variety of lipoproteins when heterodimerized with TLR1 or TLR6, which prompts inflammatory signaling in insulin-responsive cell types like adipocytes, hepatocytes, and skeletal muscle [287–289]. Kuo et al. showed that aged or diet-induced obesity in the TLR2 KO mice was resistant to adipocyte hypertrophy and adiposity while having improved glucose tolerance and insulin sensitivity compared with wildtype controls [290]. TLR2 KO mice had increased insulin-induced phosphorylation of AKT and IRS-1, indicating elevated insulin signaling in the liver [290]. Similarly, TLR4 recognizes LPS and activates inflammatory pathways through NF-kB, AP1, and interferon-regulatory factors (IRFs) [291]. Female mice that lack TLR4 are protected against insulin resistance associated with high-fat-induced obesity [204]. Male mice with adipocyte-specific deletion of TLR4 (Tadipo) had decreased TLR4 expression in the liver and acutely improved insulin sensitivity [292]. In the long term, the Tadipo mice have reduced adiponectin and insulin sensitivity [292]. Thus, this data suggests TLR4 has a dichotomous effect on adipose tissue. Mice with hepatocyte-specific KO of TLR4 (Tlr4^LKO^) fed a high-fat diet to induce obesity had improved glucose tolerance and insulin sensitivity compared with control mice [285]. Mice with a hematopoietic-specific deletion of TLR4 had enhanced insulin sensitivity in adipose and liver tissues due to reduced JNK kinase activity [293]. Ultimately, an increased flux of TLR ligands like dietary fatty acids, free fatty acids, and lipopolysaccharides contributes to insulin resistance in the adipose and liver.

From a clinical perspective, TLR4 antagonists and anti-TNFα therapies hold potential as therapeutic agents against MASLD. However, the first TLR4 antagonist tested in human clinical trials, ApTOLL, was developed for cardiovascular diseases and has not been shown to reduce MASLD or associated metabolic disorders [294]. Further research is needed to evaluate the efficacy of TLR4 antagonist treatments in patients with MASLD. Since TNFα can be induced by TLR4 activation, it represents a potential treatment target. Animal studies have demonstrated that reducing TNFα levels can lead to favorable metabolic effects [295]. Nevertheless, additional studies on their effectiveness and clinical utility in humans are needed to elucidate their potential therapeutic uses.

##### Adiponectin

Adiponectin is considered a healthy adipokine as levels are inversely associated with body weight [296]. Metabolically healthy obese individuals display adiponectin levels comparable with those with normal weight [297]. Adipocytes secrete adiponectin to stimulate fatty acid oxidation and inhibit glucose production in the liver via its receptor(s), AdipoR1 and AdipoR2 [298]. Adiponectin indirectly activates PPARα via AMPK and MAPK kinase signaling mechanisms in muscle cells to induce fatty acid oxidation, decreasing intracellular lipid content [299]. It inhibits liver gluconeogenesis by suppressing glucogenic genes *PCK1* and *G6PC* [300]. Adiponectin is also an important anti-inflammatory adipokine through mechanisms that are not fully understood. Diet-induced inflammation and insulin insensitivity were prevented in mice lacking the adaptor protein APPL2 (adaptor protein, phosphotyrosine interacting with PH domain and leucine zipper 2), which acts as a scaffold for adiponectin and adiponectin receptors, adipoR1 and adipoR2, and negatively regulates adiponectin signaling [301]. Single-nucleotide polymorphisms (SNPs) in *ADIPOR1* and *ADIPOR2* genes have been implicated in hepatic fat accumulation and insulin resistance [297,302,303]. Adiponectin binding to adipoR1 activates AMPK, and PPARα suppresses hepatic DNL activity and lipogenesis [300]. Adiponectin resistance in obesity diminishes the ability of adiponectin to maintain insulin sensitivity, blood glucose levels, and hepatic fat accumulation [298]. Adiponectin levels in patients with T2DM were inversely related to MASLD development, and the decrease in adiponectin is associated with the degree of steatosis in T2DM [304,305]. Furthermore, HSC-specific overexpression of adiponectin significantly reduced liver fibrosis in mice, independent of adiponectin from adipocytes [174]. Overall, adiponectin is considered an anti-inflammatory adipokine that promotes insulin sensitivity, and there are still many areas to be learned about how this hormone functions, especially how its loss in HSC activates fibrotic pathways, but the protein is not expressed in these cells.

### Neurons in the adipose–liver axis

#### Leptin

**Figure 8:**
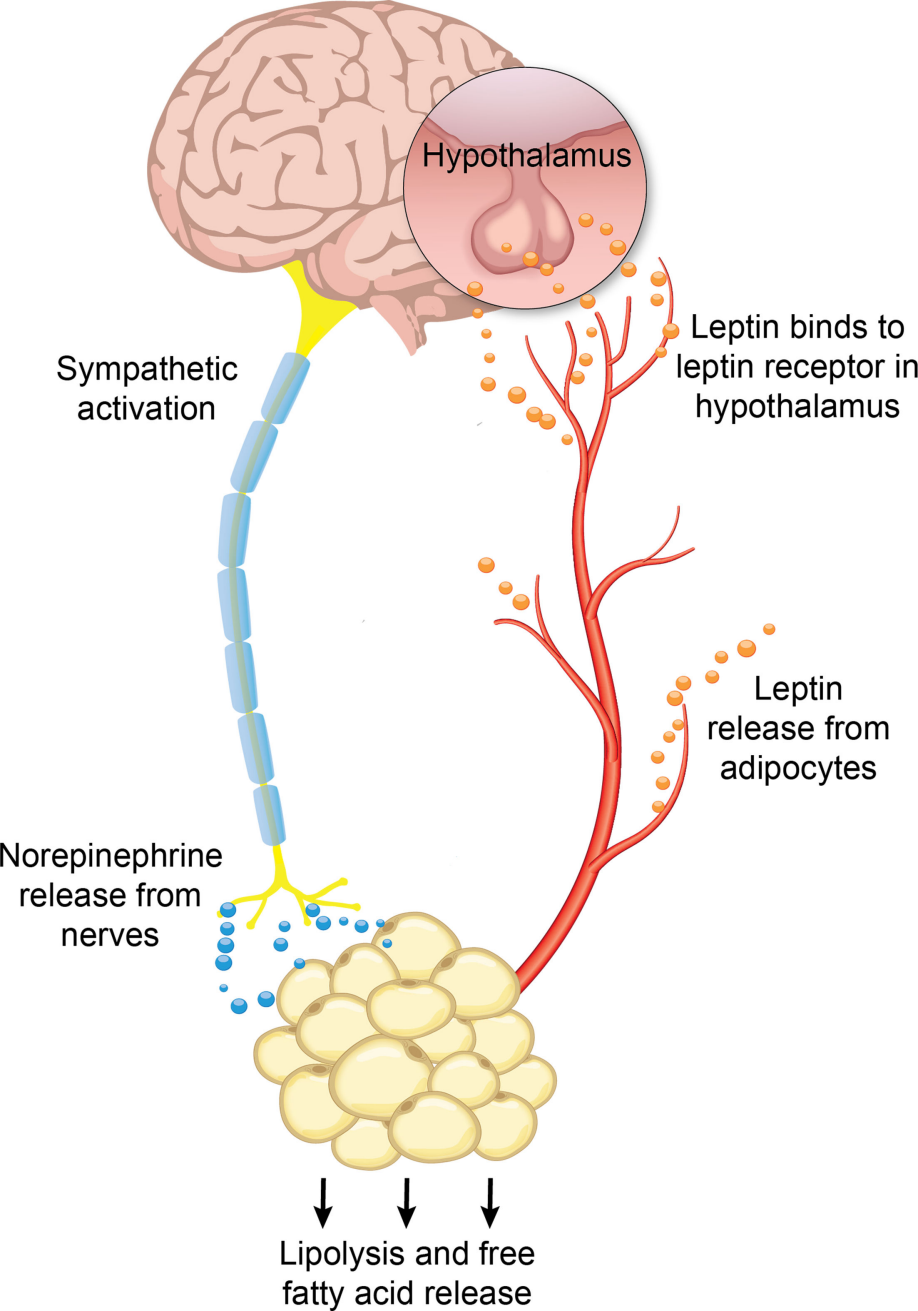
Leptin is a satiety signal released by adipose tissue into the bloodstream. Leptin binds its receptor to cross the blood–brain barrier and then acts on the hypothalamus. The hypothalamus responds by activating the sympathetic nervous system, and the nerves that innervate brown adipose tissue release norepinephrine. The norepinephrine triggers lipolysis and releases free fatty acids from the adipocytes.

Leptin is produced by adipocytes, primarily WAT, and acts on the leptin receptor (LepRb) in the hypothalamus to produce satiety effects (Figure 8). Leptin is elevated in obesity, and there is often central and peripheral leptin resistance in obese individuals, resulting in diminished energy expenditure and increased appetite [298]. Leptin binds pro-opiomelanocortin (POMC) neurons and signals the hindbrain to inhibit food intake and increase energy expenditure by inducing BAT thermogenesis [298]. Leptin binding activates the JAK2 (Janus kinase 2)/STAT3 (signal transducer and activator of transcription 3), PI3K (phosphoinositide 3-kinase)/AKT, and ERK pathways, allowing it to manage appetite, metabolism, and insulin sensitivity [298]. Leptin increases sympathetic signaling in BAT and WAT to promote lipolysis [300]. However, elevated leptin drives the up-regulation of SOCS3 (suppressor of cytokine signaling 3) and PTP1B (protein tyrosine phosphatase 1B) in central leptin resistance, negatively affecting leptin signaling by acting on JAK2 and STAT3 [306]. This process also depends on leptin’s inflammatory effects, as high leptin levels drive increases in IL-6 and IL-10, which regulate the expression of SOCS3 [306]. Leptin’s other inflammatory effects include the induction of phagocytosis and acting as an eosinophil survival factor [307]. Leptin normally prevents lipid accumulation in the liver by increasing triglyceride export and decreasing DNL activity by interacting with SCD1 and SREBP1 [308,309]. However, when there is leptin resistance in the liver and increased plasma leptin levels, there is increased expression of SREBP-1, causing lipogenesis that contributes to the development of MASLD [310]. Thus, leptin levels are associated with hepatic steatosis severity and degree of liver fibrosis [310].

Glucagon-like peptide 1 (GLP-1) and glucose-dependent insulinotropic polypeptide (GIP) are two incretins that are released from the gut and stimulate feelings of being full and reduce appetite [311]. Their receptors, GLP-1 receptor (GLP-1R) and GIP receptor (GIPR), respectively, are ubiquitously expressed but have different functions in different organs. The hypothalamic receptors induce satiety, potentially through repression of orexigenic neuropeptide Y [311]. There is a complex relationship between leptin and GLP-1. Leptin has been shown to stimulate the release of GLP-1 by activating leptin receptors on cells that secrete GLP-1 [312]. Studies have also shown that GLP-1R agonist administration decreases plasma levels of leptin, thereby improving leptin resistance, while GIPR agonism does the opposite [313]. These findings give insight into the popular GLP-1R and GIPR agonists, such as semaglutide (Ozempic) and tirzepatide (Mounjaro), and how they affect liver-adipose-brain signaling and suggest that in individuals with leptin resistance, GLP-1R agonists may be a solution.

#### Brain to adipose nerve innervation

Adipose tissue depots are innervated, more predominantly in BAT, by various nerve types, including sensory, parasympathetic, and sympathetic neurons. However, the signaling mechanisms of these neurons remain largely unidentified. The hypothalamus and BAT depot communicate with each other to maintain energy homeostasis. The hypothalamus induces sympathetic activation, resulting in the release of norepinephrine from nerves in BAT (Figure 7). This process leads to intracellular lipolysis, thereby releasing fatty acids [298]. Consequently, the hypothalamus and nerve innervation mediate lipolysis, which may contribute to MASLD by increasing the release of FFAs to the bloodstream. Additionally, sympathetic activation of BAT is a significant source of glucose uptake, as it stimulates GLUT1, potentially counteracting the development of insulin resistance [314].

Diabetic neuropathy is characterized by nerve damage in the skin caused by persistently high blood glucose levels. Due to the heterogeneity of the condition, the prevalence has been estimated to range from 10% to 90%, with approximately 50% of patients in diabetes clinics being diagnosed. It is thought to be more severe and prevalent in T2DM than in Type 1 Diabetes (T1D) [315,316]. Given the overlap between the prevalence and phenotypes of T2DM and MASLD, it is plausible that some patients with MASLD will also be experiencing neuropathies. Diabetic neuropathy is characterized by damage to nerve fibers in the skin, which may also occur in the adipose depots. Blaszkiewicz et al. demonstrated that when diabetic neuropathy occurs, adipose neuropathy can also occur [317]. They showed that in leptin-deficient mice, there was a decrease in total nerve supply, sympathetic nerve activation, and catecholamine synthesis, indicating a loss of adipose innervation and adipose neuropathy in obese and diabetic mice [317]. The same was observed in the BAT, along with tissue whitening and a decrease in UCP1, which is essential for sympathetic activation of thermogenesis [317]. These findings were mirrored in human samples taken from elective surgeries, as obese human WAT had decreased innervation [317]. The loss of sympathetic BAT innervation could decrease sympathetic-induced BAT glucose uptake, thereby further increasing blood glucose levels in patients with diabetes. In a different study, after 10 weeks of high-fat feeding to induce obesity, male mice showed almost double the sympathetic nerve activation in their livers, indicating that obesity-induced MASLD has overactive sympathetic activity [318]. However, tyrosine hydroxylase (TH)-expressing neurons are reduced in metabolic liver diseases [319]. Peripheral nerves are not only affected by MASLD, but they may also contribute to its development. It has been demonstrated that hepatic steatosis is significantly reduced following sympathetic nerve ablation with 6-hydroxydopamine or liver-specific ablation, as indicated by histological and biomarker assessments [318]. These findings suggest an underappreciated role of the peripheral and hepatic nervous systems in MASLD, both as a phenotype and a potential driver.

## Conclusions

In summary, the molecular mechanisms that initiate MASLD are intricate and not entirely comprehended. The liver–adipose tissue axis orchestrates fasting and feeding responses that become dysregulated in metabolic diseases. Several pivotal factors have been identified as mediators of hepatic insulin sensitivity, including hepatic bile acid-FXR signaling during feeding and the bilirubin–PPARα axis during fasting; when these critical pathways become dysfunctional, the progression to MASLD commences. There is limited knowledge regarding the bilirubin–PPARα axis, and additional research into the enzymes heme oxygenase, BVRA, and UGT1A1 is imperative to understand how bilirubin metabolism affects MASLD and their potential as therapeutic targets for it. The mechanisms by which adipose-derived hormones such as adiponectin and leptin signal to the liver, thereby regulating hepatic insulin sensitivity and liver fibrosis, are also essential. Pharmacological agents such as GLP-1 receptor agonists are pertinent in cases of leptin resistance, as they can enhance satiety and contribute to restoring leptin sensitivity. Factors regulating hepatic insulin turnover, including IDE and CEACAM1, are critical mediators in hepatic insulin sensitivity and can influence peripheral tissues. The potential of CEACAM1 as a druggable target for MASLD remains to be determined; further investigation is necessary. Moreover, other vital components such as the gut-liver axis, microbiome, and epigenetic modifications have also been implicated in MASLD pathogenesis. A comprehensive elucidation of the mechanisms underlying adiposity, oxidative stress, inflammation, and insulin resistance is essential for advancing our understanding of MASLD, thereby facilitating the development of more effective therapeutic strategies.

## Data Availability

This review article contains no datasets generated or analyzed during the current study except Figure 2.

## Abbreviations

ACC: acetyl-CoA carboxylase
ACOX1: acyl-CoA oxidase 1
APPL2: adaptor protein, phosphotyrosine interacting with PH domain and leucine zipper 2
AS160: AKT substrate 160
ATGL: adipose triglyceride lipase
BAT: brown adipose tissue
BMI: body mass index
BilR: bilirubin reductase
CA: cholic acid
CDCA: chenodeoxycholic acid
CDK5: cyclin-dependent kinase 5
CEACAM1: carcinoembryonic antigen cell adhesion molecule 1
COX: cyclooxygenase
CPT1A: carnitine palmitoyl-transferase 1A
ChIP-seq: Chromatin immunoprecipitation sequencing
ChREBP: carbohydrate response element-binding protein
DCA: deoxycholic acid
DGAT: diacylglycerol acyltransferase
DNL: *de novo* lipogenesis
ERK: extracellular signal-regulated kinase
FASN: Fatty acid synthase
FATP: fatty acid transport protein
FFA: free fatty acid
FGF21: fibroblast growth factor 21
FGFR1c and FGFR3c: FGF receptor 1c and 3c
FOXO1: forkhead box protein O1
FXR: farnesoid x receptor
GLUT4: glucose transporter type 4
G6PC: glucose-6-phosphatase
G6Pase: glucose-6-phosphatase
GR: glucocorticoid receptor
HDL: high-density lipoprotein
HFD: high-fat diet
HNFa: hepatocyte nuclear factor 4 alpha
H_2_O_2_: hydrogen peroxide
HSL: hormone-sensitive lipase
IDL: intermediate-density lipoproteins
IL-6: interleukin-6
IL-1β: interleukin-1b
IRFs: interferon-regulatory factors
IRS-1: insulin receptor substrate 1
JAK2: Janus kinase 2
JNK: c-Jun N-terminal kinase
KHK: ketohexokinase
KLB: b-Klotho
12/15-LO: 12/15-lipoxygenase
LPL: lipoprotein lipase
LepRb: leptin receptor
MASLD: metabolic dysfunction-associated steatotic liver disease
MASLD: metabolic dysfunction-associated steatotic liver disease
MGAT: Monoacylglycerol acyltransferase
MPK-1: MAP kinase phosphatase
NMR: nuclear magnetic resonance
NOX4: NADPH oxidase
O^-^: superoxide anions
PCK2: phosphoenolpyruvate carboxykinase 2
PEPCK: phosphoenolpyruvate carboxykinase
PFK: phosphofructokinase
PG: produce prostaglandins
PGC-1α: proliferator-activated receptor gamma coactivator 1α
PI3K: phosphoinositide 3-kinase
POMC: pro-opiomelanocortin
PPAR: peroxisome proliferator-activated receptors
PPAR: proliferator-activated receptor
PTP1B: protein tyrosine phosphatase 1B
PUFAs: polyunsaturated fatty acids
ROS: reactive oxygen species
S112A: Ser112 to alanine
SCD1: Stearoyl-CoA desaturase 1
SHP: small heterodimer partner
SNPs: single nucleotide polymorphisms
SOCS3: suppressor of cytokine signaling 3
SREBP1c: sterol regulatory element binding protein-1c
STAT3: signal transducer and activator of transcription 3
Ser112: Serine 112
Ser273: Serine 273
Smad3: small mothers against decapentaplegic 3
T1D: Type 1 Diabetes
T2DM: type 2 diabetes mellitus
T2DM: type 2 diabetes mellitus
TGFβ: transforming growth factor-β
TH: tyrosine hydroxylase
TLR: toll-like receptor
TNFα: tumor necrosis factor a
TZDs: thiazolidinediones
UCP1: uncoupling protein 1
UGT1A1: uridine diphosphate glucuronosyltransferase 1A1
VLDL: very low-density lipoprotein
WAT: white adipose tissue
iWAT: inguinal WAT
